# First decompensation outweighs portal vein thrombosis in cACLD: Molecular profiling and derivation of the VAIL score

**DOI:** 10.1016/j.jhepr.2026.102017

**Published:** 2026-08-29

**Authors:** Erica Villa, Rosina Maria Critelli, Rossella Donghia, Endrit Shahini, Fabiola Milosa, Filippo Scianò, Francesco Dituri, Gianluigi Giannelli

**Affiliations:** 1 CHIMOMO Department, University of Modena and Reggio Emilia, Modena, Italy; 2 National Institute of Gastroenterology “Saverio de Bellis”, IRCCS, Castellana Grotte (BA), Italy

**Keywords:** Portal hypertension, Portal vein thrombosis, Decompensation, Biomarkers, Endothelial dysfunction, VAIL score

## Abstract

**Background & Aims:**

The temporal relationship between hepatic decompensation and portal vein thrombosis (PVT) in compensated advanced chronic liver disease (cACLD) remains unclear. We investigated their temporal sequence, prognostic significance, and molecular signatures and developed a biologically grounded predictive model for decompensation.

**Methods:**

In this prospective study, 367 rigorously defined patients with cACLD without previous decompensation or PVT were followed for up to 11 years. Baseline assessment included hepatic venous pressure gradient, liver stiffness, upper endoscopy, Doppler ultrasound, and serum measurements of 35 biomarkers spanning angiogenesis, inflammation, endothelial injury, hypoxia, and gut permeability. Temporal associations were analysed using Kaplan-Meier and time-dependent Cox models. Biomarkers independently associated with decompensation were integrated to derive the VAIL (VEGF-D, Angiopoietin-1, I-FABP, IL-6) score, which was compared with established prognostic scores.

**Results:**

During follow-up, 102/367 (27.7%) patients developed hepatic decompensation, and 22/367 (6.0%) developed non-malignant PVT. Among patients experiencing both events, decompensation preceded PVT in 53.3%, whereas PVT preceded decompensation in 33.3%. Time-dependent Cox analysis showed that hepatic decompensation was the strongest determinant of mortality (hazard ratio [HR] 11.86, 95% CI 5.68–24.78; *p* <0.001), whereas PVT was not independently associated with mortality (HR 0.56, 95% CI 0.12–2.46; *p* = 0.443). Biomarker profiling showed a restricted signature for isolated PVT (Tie2 and mesothelin) but broad multisystem dysregulation preceding decompensation. The VAIL score showed the most robust predictive performance (optimism-corrected coefficient 1.76, 95% CI 1.48–4.36; *p* <0.001) after bootstrap validation, outperforming all established prognostic scores.

**Conclusions:**

In cACLD, hepatic decompensation is an early, biologically active, and prognostically dominant event, whereas PVT is a late, biologically restricted complication. The VAIL score provides superior mechanism-based risk stratification for predicting clinical deterioration.

**Impact and implications:**

This study provides prospective evidence that hepatic decompensation represents an early, biologically active, and prognostically dominant transition in compensated advanced chronic liver disease, whereas portal vein thrombosis arises later and reflects a distinct process with limited independent prognostic impact. These findings are important for clinicians and researchers as they clarify the temporal and biological hierarchy of key disease events, helping refocus risk assessment on pathways driving decompensation rather than on downstream vascular complications. The identification of reproducible biomarker signatures and the derivation of the VAIL score suggest that biomarker-informed risk stratification could support earlier identification of patients at high risk of decompensation and guide preventive strategies in clinical practice. Although confirmation in multicentre cohorts is required, this work provides a biologically grounded framework for future studies to test whether targeted, biomarker-guided interventions can modify the disease trajectory in advanced chronic liver disease.

**Clinical Trials number:**

ClinicalTrials.gov (NCT03083002).

## Highlights

• Hepatic decompensation is an early, biologically active transition in cACLD.
• Portal vein thrombosis emerges later with limited independent prognostic impact.
• Decompensation is preceded by multisystem angiogenic, inflammatory, and hypoxic dysregulation.
• The VAIL score accurately predicts first decompensation using biologically grounded markers.
• Internal validation confirms robust and stable discrimination across time horizons.

## Graphical abstract

**Figure:**
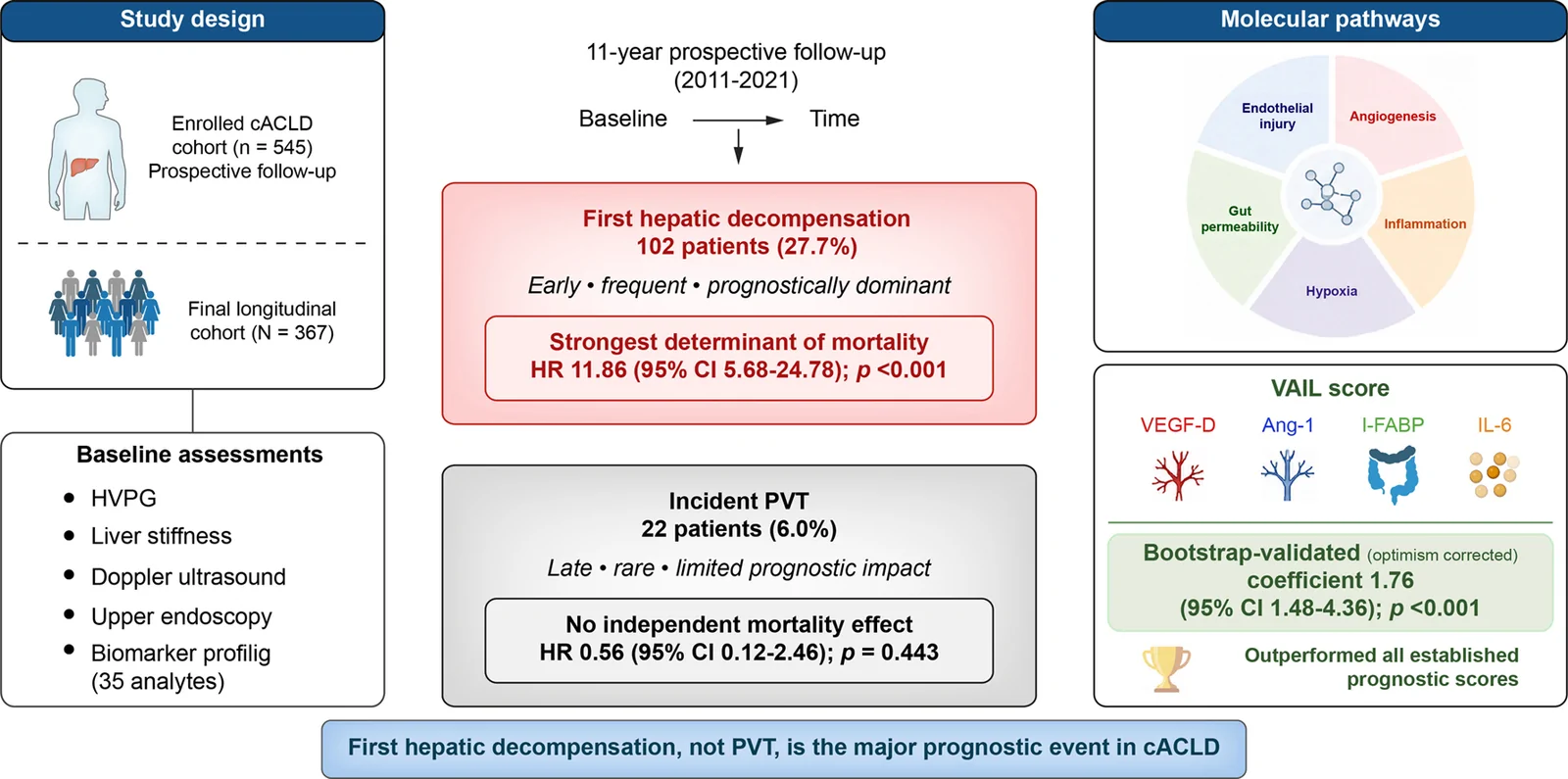

## Introduction

Portal vein thrombosis (PVT) and hepatic decompensation are pivotal events in the natural history of chronic liver disease (CLD), often marking the transition from compensated to decompensated cirrhosis. Both conditions are associated with increased morbidity and healthcare utilization.^1^^,^^2^ Although mortality is clearly linked to the occurrence of decompensation, the prognostic impact of PVT remains debated.^1^^,^^3^^,^^4^ The temporal sequence, clinical relationship, and underlying pathophysiological connections between PVT and decompensation are still poorly defined.[1], [2], [3]

PVT has traditionally been regarded as a consequence of portal hypertension (PH) and reduced portal flow, potentially worsening disease progression through intestinal congestion, systemic inflammation, and impaired hepatic perfusion.^5^ In contrast, hepatic decompensation—manifesting as ascites, variceal bleeding, or hepatic encephalopathy—reflects progressive hepatic dysfunction and worsening circulatory derangements.[6], [7], [8] Proposed contributors to both complications include endothelial dysfunction, procoagulant imbalance, gut barrier disruption, and low-grade systemic inflammation. Prospective data evaluating their interrelationship and shared molecular drivers are scarce. Despite overlapping clinical features, it remains unclear whether one event typically precedes the other or whether both arise in parallel through shared mechanisms.

Because hepatic decompensation is the principal determinant of mortality in compensated advanced chronic liver disease (cACLD) and current prognostic scores do not capture its key biological drivers, we also aimed to develop a mechanistically informed predictive score for first decompensation. This approach was intended to integrate vascular, angiogenic, inflammatory, and liver-specific pathways that more accurately reflect the early biological shifts preceding clinical deterioration.

This study therefore sought to (1) define the sequence in which PVT and decompensation occur; (2) identify clinical and haemodynamic predictors of each event; (3) characterize the biomarker profiles associated with their development; and (4) derive a biologically grounded predictive score for first decompensation. A clearer understanding of these temporal and mechanistic links may refine risk stratification and inform preventive strategies.

The primary objective of the study was to define the temporal and prognostic relationship between incident non-malignant PVT and first hepatic decompensation in patients with rigorously defined cACLD. Because first decompensation emerged as the dominant clinical transition, we further sought to identify baseline biological signatures associated with this event and to derive a candidate biomarker-based score for first decompensation**.**

## Patients and methods

### Study design and population

We conducted a prospective cohort study at the Gastroenterology Unit of the University of Modena between January 2011 and October 2021. A total of 545 consecutive adult patients with cACLD were enrolled.

Inclusion criteria were age 18–75 years; cirrhosis of any aetiology diagnosed using clinical, biochemical, and elastographic criteria; and absence of hepatocellular carcinoma (HCC) at baseline. Exclusion criteria included prior liver transplantation, HCC at enrolment or within 6 months, HIV coinfection, and inability to complete follow-up. All participants provided written informed consent.

### Baseline assessment

At enrolment, patients underwent standardized clinical, haemodynamic, imaging, and laboratory assessment:

• upper gastrointestinal endoscopy to grade oesophageal varices according to Baveno VI criteria;^9^
• abdominal ultrasound and liver stiffness measurement (LSM) via transient elastography (FibroScan®, Echosens, Paris, France);
• hepatic venous pressure gradient (HVPG) measurement using a balloon-tipped catheter under conscious sedation, with triplicate readings obtained by experienced operators; and
• routine laboratory testing, including liver biochemistry, complete blood count, coagulation profile, and alpha-foetoprotein (AFP). Peripheral blood was collected at baseline, processed within 2 h, aliquoted, and stored at -80 °C.

### Biomarker profiling and development of the VAIL score

To identify biologically relevant predictors of hepatic decompensation, we performed comprehensive baseline biomarker profiling in all participants. Serum concentrations of 35 predefined proteins were quantified in duplicate using a multiplex Ella ELISA platform (Bio-Techne srl, Milan, Italy), covering inflammation (*e.g.* CRP, IL-6, and TNF-α), immune modulation (*e.g.* CD14 and IL-10), hypoxia-related stress (*e.g.* I-FABP and CA9), coagulation (*e.g.* vWF and TPO), and angiogenesis (*e.g.* Angiopoietin-1, Angiopoietin-2, ANGPTL4, and VEGFA). Repeat biomarker assessment was performed in 237/345 patients (68.7%) still alive at ∼2–4 years and in 218/305 patients (71.4%) still alive at 6 years. The remaining patients did not have repeat measurements because of sample unavailability or loss to follow-up. Repeat measurements were analysed descriptively to assess persistence or progression of biological dysregulation and were not incorporated into the primary VAIL (VEGF-D, Angiopoietin-1, I-FABP, IL-6) model. The complete biomarker panel is listed in the Supplementary Materials and methods.

The VAIL score was derived exclusively from baseline biomarker values measured at enrolment. To derive a multivariable risk model, we first screened biomarkers associated with incident hepatic decompensation using Cox proportional hazards models. Z-score-standardized biomarkers were then entered into a least absolute shrinkage and selection operator (LASSO) Cox regression model for variable selection and regularization, with coefficients of less informative variables shrunk towards zero. Biomarker values were log-transformed to reduce skewness and standardized; retained predictors were combined into a weighted score using their β-coefficients.

Model performance was evaluated using time-dependent receiver operating characteristic (ROC) curves, internal cross-validation, and 1,000 bootstrap resamples at 3- and 10-year time points. Discrimination and calibration were compared against established clinical and fibrosis-based prognostic indices, including Child-Pugh,^10^ model for end-stage liver disease (MELD),^11^ early prediction of decompensation (EPOD),^12^ AST-to-platelet ratio index (APRI),^13^ albumin–bilirubin (ALBI),^14^ fibrosis-4 (FIB-4),^15^ von Willebrand factor antigen-to-platelet ratio (VITRO),^16^ and Chronic Hepatic Event Stratification System–ALARM (CHESS-ALARM).^17^

### Statistical analysis

#### Follow-up and outcomes

Patients were followed every 6–12 months with clinical assessment, laboratory testing, and imaging (ultrasound, CT, or MRI), including HCC surveillance in accordance with EASL guidelines.^18^ Portal vein patency was systematically assessed during scheduled imaging follow-up every 6 months using Doppler ultrasound. Portal flow direction and portal vein velocity were recorded when technically feasible. Suspected PVT was confirmed in all cases by contrast-enhanced CT or MRI, both to define thrombus extension and to exclude malignant portal vein obstruction related to HCC. PVT was defined by the presence of echogenic material within the portal venous lumen. PVT was classified using a treatment-oriented anatomical–occlusive approach. For each event, we recorded chronicity, degree of luminal obstruction, anatomical extension within the portomesenteric axis, presence of cavernous transformation, and interval evolution on follow-up imaging. Complete main PVT was defined as the absence of residual lumen in the main portal vein. Extension to the splenic vein, portal confluence, and/or superior mesenteric vein was recorded separately. In patients receiving anticoagulation, the response was categorized as complete recanalization, partial recanalization/regression, stable thrombosis, or progression.

Primary outcomes were incident PVT, hepatic decompensation (ascites, variceal haemorrhage, or hepatic encephalopathy), and HCC. Event dates were prospectively recorded.

#### Descriptive statistics

Continuous variables were reported as mean ± SD or median (IQR), as appropriate. Categorical variables were summarized as frequencies and percentages. Between-group comparisons were performed using Student’s *t* test or the Mann-Whitney *U* test for continuous variables, and χ^2^ or Fisher’s exact tests for categorical variables.

#### Time-dependent and competing-risk analyses

To assess the temporal interplay and prognostic impact of decompensation, PVT, and HCC, we constructed time-dependent Cox proportional hazards regression models with all-cause mortality as the outcome. Each event was modelled as a time-varying covariate, contributing dynamically to mortality risk from the time of its occurrence. Time was structured using the counting process (start–stop format), and models were adjusted for age, sex, MELD score, and relevant baseline covariates. Fine–Gray competing-risk models were not used because of the complexity of sequential and overlapping events.

The cumulative incidence of first clinical events (hepatic decompensation, PVT, HCC, and death) was estimated using the Aalen–Johansen estimator, accounting for competing risks. Event categories were mutually exclusive and defined by the first event occurring during follow-up, with death treated as a competing event for non-fatal outcomes.

#### Time-to-event analysis

Event-free survival was estimated using Kaplan-Meier curves and compared using the log-rank test. Cox proportional hazards regression was used to estimate hazard ratios (HRs) and 95% CIs; *p* <0.05 was considered statistically significant. Kaplan-Meier methods were used exclusively for overall survival analyses and not for estimation of cumulative incidence in the presence of competing risks.

Cox models were used to identify baseline predictors of short-term (3-year) and long-term (10-year) risk of hepatic decompensation, and long-term risk of decompensation with PVT. Short-term prediction of PVT was not assessed because of the low number of early events. Candidate covariates included age, sex, aetiology, BMI, diabetes, HVPG, portal vein velocity (Vm), LSM, large varices, AST, ALT, bilirubin, platelets, albumin, creatinine, and international normalized ratio (INR). To avoid collinearity, each component of composite scores was modelled individually. Because aetiologic treatment was strongly determined by disease aetiology and treatment era, it was not modelled as an independent predictor; instead, aetiology was included among candidate baseline covariates, and treatment distribution by aetiologic subgroup is presented in Table 1. As treatment exposure during follow-up was dynamic, non-randomized, and clinically indicated, it was not included as an independent causal predictor in the primary models. Instead, we performed an exploratory association analysis to evaluate relationships among follow-up treatments, baseline large oesophageal varices, and incident hepatic decompensation, using the χ^2^ test or Fisher’s exact test, as appropriate. A secondary multivariable model incorporated all established prognostic scores (ALBI, APRI, CHESS-ALARM, Child-Pugh, EPOD, FIB-4, MELD, and VITRO) alongside the newly proposed VAIL score. Variables with *p* <0.10 in univariable analyses were included in multivariable models.

**Table 1:** Baseline characteristics of the final longitudinal cohort of 367 patients with compensated advanced chronic liver disease included in the primary analysis.

Characteristic	Final cohort (N = 367)	Recompensated (n = 162)	*p* value	No prior PVT (n = 502)	Prior PVT (n = 43)	*p* value
**Demographics**						
Age (years)	58.2 ± 11.7	59.9 ± 9.4	0.112	58.8 ± 11.2	56.6 ± 10.5	0.190
Male sex, n (%)	230 (62.7)	122 (75.3)	0.004	331 (65.9)	30 (69.8)	0.610
BMI (kg/m^2^)	27.4 ± 4.9	26.7 ± 5.0	0.044	27.1 ± 5.0	26.3 ± 4.8	0.370
Aetiology of liver disease, n (%)						
Viral hepatitis	181 (49.3)	56 (34.0)		229 (45.6)	15 (34.9)	
HBV	37 (20.4)	11 (19.6)		48 (21.0)	4 (26.7)	
HCV	144 (79.6)	45 (80.4)		181 (79.0)	11 (73.3)	
Alcohol-related	59 (16.1)	66 (40.7)	<0.001	113 (22.5)	11 (25.6)	0.178
NASH	68 (18.5)	30 (18.5)		91 (18.1)	9 (20.9)	
Other	59 (16.1)	11 (6.8)		69 (13.7)	8 (18.6)	
Antiviral treatment, n (%)						
HBV analogues	36 (97.2)	11 (100)	0.260	48 (100)	3 (75.0)	0.576
Viral suppression	36 (100)	10 (90.9)	0.899	46 (95.8)	3 (100)	0.998
HCV DAAs	95 (65.9)	31 (68.8)	0.718	121 (66.8)	7 (63.6)	0.810
SVR	94 (98.9)	31 (100)	0.415	119 (98.3)	6 (85.7)	0.732

**Clinical and laboratory variables**						
MELD score	9.3 ± 3.8	11.5 ± 4.0	<0.001	9.9 ± 4.3	9.7 ± 3.6	0.795
Child-Pugh class, n (%)						
A	332 (90.5)	105 (64.8)	<0.001	417 (83.1)	33 (76.7)	0.294
B	35 (9.5)	57 (35.2)		85 (16.9)	10 (23.3)	
HVPG	12.8 ± 7.1	18.8 ± 6.2	<0.001	14.6 ± 7.3	15.5 ± 8.2	0.431
LSM	19.2 ± 15.0	26.9 ± 18.6	<0.001	21.2 ± 16.0	23.9 ± 21.4	0.558
Platelet count ( × 10^9^/L)	131 ± 64	101 ± 64	<0.001	124 ± 77	107 ± 80	0.189
Albumin (g/dl)	3.8 ± 0.5	3.4 ± 0.5	<0.001	3.7 ± 0.6	3.5 ± 0.5	0.005
Bilirubin (mg/dl)	1.3 ± 2.2	2.1 ± 2.0	<0.001	1.5 ± 2.2	1.8 ± 2.0	0.339
INR	1.2 ± 0.2	1.4 ± 0.3	<0.001	1.2 ± 0.3	1.2 ± 0.2	0.306
Creatinine (mg/dl)	0.9 ± 0.8	0.9 ± 0.4	0.400	0.9 ± 0.6	0.9 ± 0.3	0.904
Presence of large varices, n (%)	28 (7.6)	20 (14.8)	0.015	48 (9.6)	9 (20.9)	0.019

**Imaging findings**						
Portal vein diameter (mm)	12.8 ± 2.5	14.9 ± 6.9	0.064	13.3 ± 7.0	14.3 ± 6.3	0.405
Portal flow velocity (cm/s)	24.4 ± 12.0	22.5 ± 7.6	0.038	24.3 ± 11.0	22.4 ±10.6	0.328

**Outcomes during follow-up**						
Incident decompensation, n (%)	102 (27.7)	154 (95.1)	<0.001	236 (47.0)	36 (83.7)	<0.001
Non-malignant PVT occurrence, n (%)	22 (5.9)	29 (17.9)	<0.001	63 (12.5)	38 (88.4)	<0.001
Incident malignant portal vein obstruction, n (%)	11 (2.9)	1 (0.6)	0.142	10 (2.0)	2 (4.7)	0.254
HCC occurrence, n (%)	62 (16.9)	24 (14.8)	0.736	86 (17.1)	7 (16.3)	0.877
Death, n (%)	112 (30.5)	71 (43.8)	<0.001	183 (36.5)	18 (41.9)	0.481

Continuous variables were compared using Student’s *t* test or the Mann-Whitney *U* test, as appropriate. Categorical variables were compared using the χ^2^ test or Fisher’s exact test, as appropriate. Reported *p* values refer to comparisons between the indicated groups; statistical significance was defined as *p* <0.05. HCC, hepatocellular carcinoma; HVPG, hepatic venous pressure gradient; INR, international normalized ratio; LSM, liver stiffness measurement; MELD, model for end-stage liver disease; NASH, non-alcoholic steatohepatitis; SVR, sustained virologic response.

#### Internal validation and split-sample sensitivity analysis

Model robustness and internal validity were primarily assessed using bootstrap resampling (1,000 iterations). In each iteration, the multivariable Cox model was re-estimated and applied to the original dataset to obtain optimism-corrected estimates of model performance.

In addition, a split-sample sensitivity analysis was performed to evaluate the stability of discrimination across independent subsamples. The full cohort (n = 367) was randomly divided into a derivation cohort (n = 200) and a validation cohort (n = 167). Discriminative performance of the VAIL score and established prognostic scores was assessed separately in the derivation and validation cohorts using time-dependent ROC curves at 3- and 10-year time points. This split-sample analysis was considered secondary and did not replace bootstrap-based internal validation.

#### Ethics and software

The study protocol was approved by the Ethics Committee of the Area Vasta Emilia Nord (AVEN) (239/12_CE_UniRer_MO) and registered at ClinicalTrials.gov (NCT03083002).

The analyses were conducted using Stata Statistical Software (Release 19, StataCorp LLC, College Station, TX, USA) and RStudio (R Foundation for Statistical Computing, Vienna, Austria).

## Results

### Study population

Of 545 enrolled patients, 43 (7.9%) had prevalent PVT. One hundred and sixty-two (29.7%) (including 27 with prevalent PVT) had a prior history of hepatic decompensation despite meeting criteria for compensated disease at the time of study enrolment. These individuals were excluded from the primary longitudinal analysis, yielding a final longitudinal cohort of 367 patients (67.3%) with rigorously defined cACLD who were prospectively followed (Table 1 and Fig. S1). This cohort represents one of the largest well-characterized prospective samples with simultaneously collected HVPG, LSM, portal flow metrics, endoscopy, imaging, and deep biomarker profiling.

### Temporal relationship between the occurrence of decompensation and PVT

During a median follow-up of nearly a decade, 102 patients (27.7%) developed hepatic decompensation. Among patients with first decompensation, abdominal imaging using Doppler ultrasound was performed at admission or within the following 3 days in all cases. The median interval between the most recent portal vein imaging and first decompensation was 5 months (IQR 2–7). Isolated decompensation was the dominant clinical trajectory, with a 120-month cumulative incidence of 24.4%, whereas decompensation associated with non-malignant PVT had a 120-month cumulative incidence of 4.2%. Overall, 22 patients developed incident non-malignant PVT; detailed PVT characteristics are reported in Table S1. The diagnosis was confirmed by CT or MRI in all cases.

Aalen–Johansen competing-risk analyses showed a progressive accumulation of first events over time (Fig. 1). Hepatic decompensation was the most frequent first event, with cumulative incidences of 15.0% at 36 months, 18.9% at 60 months, and 24.4% at 120 months. Isolated non-malignant PVT as the first event was uncommon (0.6% at 36 months, 1.1% at 60 months, and 2.5% at 120 months). Simultaneous PVT and decompensation as the first event was also rare early on but increased over time (0.6% at 36 months, 1.4% at 60 months, and 4.2% at 120 months). For context, the competing-risk cumulative incidence of HCC was 4.5%, 5.2%, and 9.4%, and that of death without a prior recorded event occurred in 2.5%, 4.2%, and 9.5% over the same time points.

**Fig. 1:**
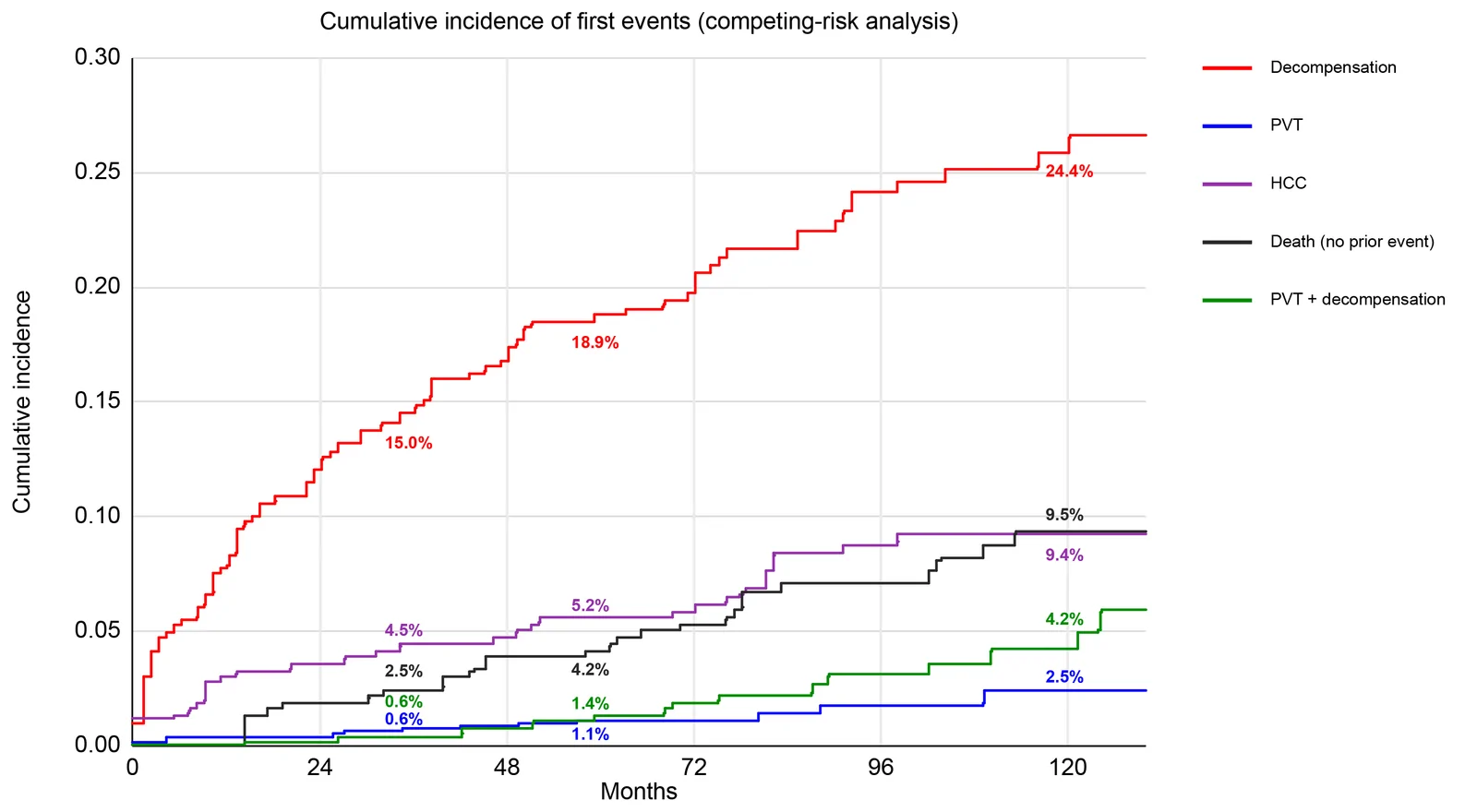
**Cumulative incidence of first clinical events in cACLD.** Cause-specific cumulative incidence functions are shown for hepatic decompensation, non-malignant PVT, HCC, death without a prior recorded event, and simultaneous PVT plus decompensation. Event categories were mutually exclusive and based on the first event observed during follow-up. Cumulative incidence functions were estimated using the Aalen–Johansen method, with death treated as a competing event for non-fatal outcomes. Percentages on the curves indicate cumulative incidence at 36, 60, and 120 months. No formal between-curve statistical comparison was performed. cACLD, compensated advanced chronic liver disease; HCC, hepatocellular carcinoma; PVT, portal vein thrombosis.

### Sequence of events: Decompensation usually comes first

Fifteen patients experienced both decompensation and PVT. In five patients (33.3%) PVT preceded decompensation, in two patients (13.3%) the two events occurred simultaneously, and in eight patients (53.3%) PVT followed decompensation. Time-to-event distributions confirmed that decompensation occurs earlier and with wider temporal variability, whereas PVT appears later and within a narrower window, consistent with being a secondary complication of advanced PH and systemic deterioration (Fig. S2).

### Biomarker profiles, decompensation, and PVT

Patients who developed hepatic decompensation—regardless of whether PVT occurred later—exhibited broad perturbation across multiple mechanistic pathways already at baseline, involving the following: angiogenesis: Angpt1 ↓, Angpt2 ↑, VEGF-D ↑; endothelial dysfunction: Nectin-2 ↑, Endoglin ↑; fibrogenesis: HA ↑, TGF-β1 ↑; inflammation: IL-6 ↑, IL-8 ↑, TNF-α ↑; hypoxia and metabolic stress: CA9 ↑; and gut barrier failure: I-FABP ↑.

These changes were present even in patients experiencing early decompensation (≤3 years), highlighting a biological vulnerability years before clinical deterioration.

Baseline biomarker profiling revealed distinct molecular signatures in patients who developed isolated hepatic decompensation compared with those with isolated PVT. In the latter group, only Tie2 and mesothelin were significantly elevated relative to patients who did not develop PVT, supporting the view that PVT is a late, flow-related complication lacking a strong upstream biological signature. In patients who decompensated later during follow-up, other additional proteins were already altered at baseline (Fig. 2). Among the 367 patients included in the final longitudinal cohort, repeat biomarker assessment showed persistent and, for several markers (particularly Angiopoietin-1, IL-6, I-FABP, VEGF-D, CA9, HA, TPO, and selected matrix remodelling markers), progressive dysregulation among patients who subsequently developed first decompensation. This pattern reflects an evolving milieu of hypoxia, endothelial injury, inflammation, and microvascular dysfunction, consistent with progression towards decompensation. Taken together, these results indicate that decompensation, rather than PVT, is biologically active, with a broad upstream signature detectable well before clinical transition.

**Fig. 2:**
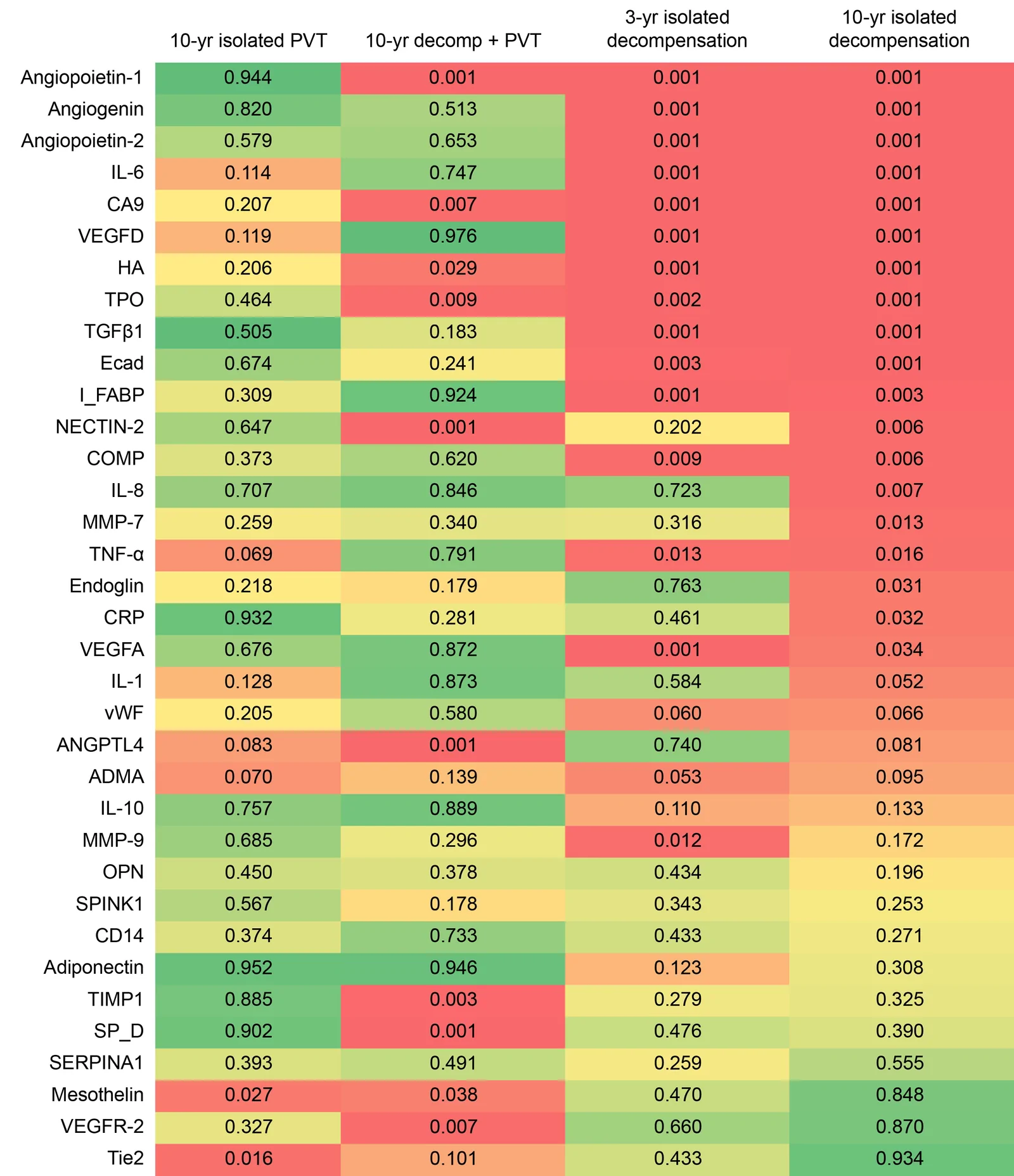
**Baseline biomarker associations with clinical outcomes**. Associations between baseline circulating biomarkers and clinical outcomes were evaluated using nonparametric tests (Mann-Whitney *U* test). Values of *p* for associations with isolated PVT, hepatic decompensation, and decompensation occurring in association with PVT at predefined time horizons are reported. Colour gradients indicate the strength of association (red = *p* <0.01; yellow = 0.01≤ *p* <0.05; green = *p* ≥0.05). FDR correction was applied to account for multiple comparisons. FDR, false discovery rate; PVT, portal vein thrombosis.

### Derivation of the VAIL score

Based on the strength and consistency of associations with incident decompensation (HR magnitude, *p* <0.05), four biomarkers were selected to construct a biologically grounded composite risk index: VEGF-D, Angiopoietin-1, I-FABP, and IL-6. These markers represent complementary mechanistic domains—vascular, angiogenic, intestinal, and inflammatory pathways—implicated in the transition from compensation to decompensation in cACLD. Their combined and pathway-level relevance formed the basis for developing the VAIL score (**V**EGF-D, **A**ngiopoietin-1, **I**-FABP, and I**L**-6).

The VAIL score was defined as follows:

(1) $$\text{VAIL}\;\text{score}=0.299{\cdot Z}_{\text{VEGF}-D}-0.803{\cdot Z}_{\text{Angpt}-1}+0.315\cdot Z_{I-\text{FABP}}+0.371\cdot Z_{\text{IL}-6}$$

where Z_X_ denotes the z-score of biomarker X:

(2) $$Z_{X}=\left[\log_{10}\left(X\right)-\mu_{\text{logX}}\right]/\sigma_{\text{logX}}$$

All biomarkers were log-transformed to reduce skewness and then standardized using the cohort-specific mean and SD of the log-transformed values.

The score was subsequently calibrated and internally validated using penalized Cox regression, time-dependent ROC curves, and bootstrap resampling. Bootstrap internal validation confirmed the robustness of the multivariable model. Among all evaluated prognostic scores, the VAIL score showed the most stable and reproducible association with decompensation, with an optimism-corrected coefficient of 1.76 and a 95% CI of 1.48-4.36 (*p* <0.001). In contrast, effect estimates for Child-Pugh, MELD, EPOD, APRI, ALBI, FIB-4, and VITRO were attenuated and no longer statistically significant after resampling, indicating limited reproducibility (Table S2). These findings demonstrate minimal overfitting and strong internal validity for VAIL (Table S2).

### Predictors of decompensation and PVT

We evaluated predictors of hepatic decompensation, considering events occurring without PVT (Table 2) and those occurring with PVT (Table S3), at both short-term (≤3 years) and long-term (≤10 years) follow-up. Across both horizons, several baseline variables were associated with decompensation, regardless of PVT status. Many of these variables were tightly linked to PH and showed substantial collinearity, reflecting shared pathophysiologic mechanisms and restricting their simultaneous inclusion in multivariable models.

**Table 2:** Baseline predictors of incident isolated hepatic decompensation in patients with compensated advanced chronic liver disease.

Variables	≤3 years				≤10 years			
	Univariate		Multivariate		Univariate		Multivariate	
	HR (95% CI)	*p* value	HR (95% CI)	*p* value	HR (95% CI)	*p* value	HR (95% CI)	*p* value
**Model 1**								
Age	1.026	0.026			1.013	0.202		
Sex^∗^	0.686	0.171			0.677	0.106		
Aetiology^†^	2.439	0.001			1.310	0.230		
BMI	1.002	0.955			1.012	0.649		
Diabetes	0.841	0.590			1.310	0.205		
HVPG	1.102	<0.001			1.100	<0.001		
Vm	0.925	0.001			0.924	<0.001		
LSM	1.028	<0.001			1.029	<0.001		
Large varices	3.767	<0.001	2.335	<0.001	4.272	<0.001	2.240	<0.001
AST	1.002	0.343			1.003	0.056		
ALT	0.996	0.835			1.000	0.915		
Bilirubin	1.060	0.037	0.983	0.666	1.074	0.003	0.971	0.506
Platelets	0.985	<0.001			0.985	<0.001		
Albumin	0.482	<0.001	0.489	<0.001	0.440	<0.001	0.493	<0.001
Creatinine	0.933	0.761			0.996	0.977		
INR	2.424	0.007	1.832	0.148	3.797	<0.001	4.030	<0.001
CRP	1.134	0.023			1.188	<0.001		

**Model 2**								
ALBI	2.423	<0.001	7.126	0.216	2.698	<0.001	4.088	0.159
APRI	1.120	0.003	0.936	0.871	1.158	<0.001	1.664	0.068
CHESS-ALARM	2.135	<0.001	1.675	0.151	1.949	<0.001	1.409	0.345
Child-Pugh class	1.922	<0.001	2.634	0.018	2.191	<0.001	2.644	0.004
EPOD	1.104	<0.001	0.572	0.320	1.068	<0.001	0.599	0.321
FIB-4	1.146	<0.001	0.861	0.379	1.163	<0.001	0.695	0.008
MELD	1.109	<0.001	1.003	0.971	1.154	<0.001	1.113	0.070
VAIL	4.949	<0.001	5.519	<0.001	3.758	<0.001	3.695	<0.001
VITRO	1.002	0.003	1.009	0.166	1.002	0.005	1.012	0.060

Cox proportional hazards regression models were used to identify baseline predictors of hepatic decompensation during follow-up. HRs with 95% CIs are reported. Variables with *p* <0.10 in univariable analyses were entered into multivariable models. Values of *p* were obtained using Wald tests. Statistical significance was defined as *p* <0.05. To minimize collinearity, components of composite scores were modelled individually. Two multivariable approaches were applied: (1) a clinical model including portal hypertension-related variables (with large oesophageal varices selected among HVPG, LSM, and portal vein velocity based on the strongest univariable association), together with bilirubin, albumin, and INR; and (2) a score-based model including established prognostic indices (Child-Pugh, MELD, ALBI, APRI, FIB-4, EPOD, CHESS-ALARM, and VITRO) alongside the VAIL score. Variables reflecting portal hypertension (HVPG, LSM, portal vein velocity, and presence of large oesophageal varices) were highly collinear and were therefore not included simultaneously in the multivariable model. Among these variables, the presence of large oesophageal varices showed the strongest univariable association with hepatic decompensation and was selected for inclusion in the primary multivariable model, together with bilirubin, albumin, and INR. ^∗^Female as reference. ^†^Non-viral aetiology as reference. ALBI, albumin–bilirubin; ALT, alanine aminotransferase; APRI, AST-to-platelet ratio index; AST, aspartate aminotransferase; CHESS-ALARM, Chronic Hepatic Event Stratification System–ALARM; CRP, C-reactive protein; EPOD, early prediction of decompensation; FIB-4, Fibrosis-4; HR, hazard ratio; HVPG, INR, international normalized ratio; LSM, liver stiffness measurement; MELD, model for end-stage liver disease; ROC, receiver operating characteristic; VAIL, VEGF-D, Angiopoietin-1, I-FABP, IL-6; VITRO, von Willebrand factor antigen-to-platelet ratio.

To address collinearity among PH-related variables, we structured the analysis into two complementary models. In model 1, among Vm, LSM, large varices, and HVPG, large varices showed the strongest univariable association with decompensation and were therefore incorporated into the multivariable model, along with bilirubin, albumin, and INR (Table 2). Model 2 evaluated the principal prognostic scores proposed for predicting first decompensation in cACLD. All scores were significantly associated with decompensation in univariable analyses.

One result was particularly consistent and compelling: the VAIL score emerged as the strongest and most informative predictor of decompensation, outperforming all individual clinical variables and all other prognostic scores. This superiority was evident at both short- and long-term follow-up and for both endpoints: decompensation alone and decompensation with PVT.

Importantly, results were almost identical whether decompensation occurred alone or in association with PVT, indicating that PVT did not confer an independent or distinct risk pattern. The consistent dominance of the VAIL score across all analyses highlights its value as a comprehensive predictor of clinical deterioration in cACLD (Table 2 and Table S3).

Classical clinical scores such as Child-Pugh retained prognostic relevance but were markedly inferior to VAIL. MELD was not independently associated with the risk of decompensation; however, it remained clinically informative. Patients who remained compensated had the lowest MELD values (8.5 ± 3.4), whereas those who developed decompensation, either alone (11.5 ± 4.8) or with PVT (11.5 ± 4.0), had significantly higher scores (both *p* <0.05). By contrast, patients who developed isolated PVT had MELD values (8.0 ± 1.8) comparable to those of stable patients and clearly lower than those of patients who decompensated (Fig. S3).

Because treatment exposure during follow-up was dynamic, non-randomized, and clinically indicated, it was not included as an independent causal predictor in the primary models. Instead, we performed an exploratory association analysis evaluating relationships between follow-up treatments, baseline large oesophageal varices, and incident hepatic decompensation, using the χ^2^ test or Fisher’s exact test, as appropriate (Table S4).

#### ROC analysis

Time-dependent ROC curves confirmed that the VAIL score outperformed all other scores at both 3 and 10 years and for both decompensation alone and decompensation with PVT (Fig. 3).

**Fig. 3:**
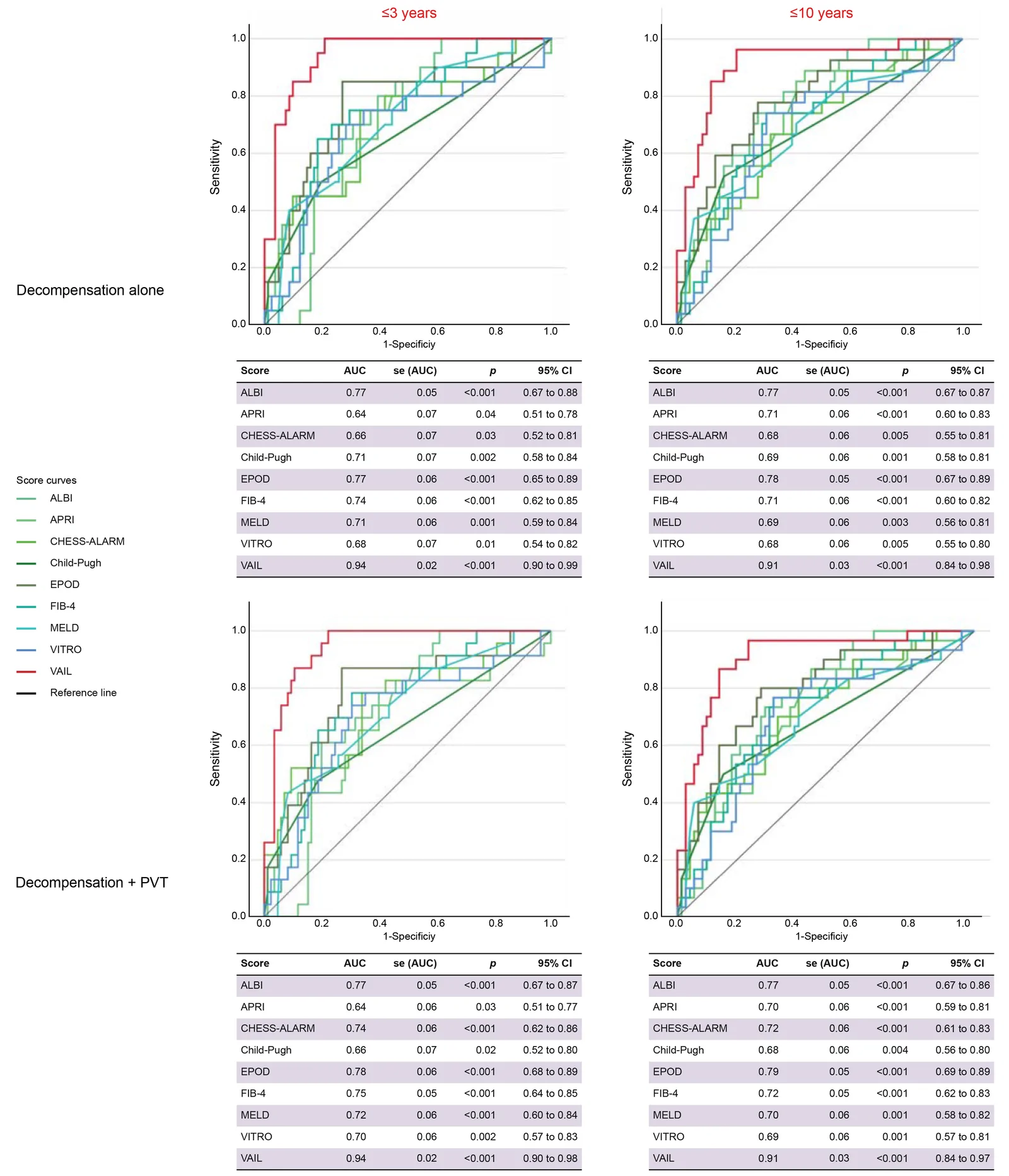
**Discriminative performance of prognostic scores for hepatic decompensation.** ROC curves comparing the ability of the VAIL score and established prognostic indices (Child-Pugh, MELD, EPOD, APRI, ALBI, FIB-4, CHESS-ALARM, and VITRO) to predict incident hepatic decompensation alone (upper panels) and decompensation associated with PVT (lower panels). Analyses were performed at ≤3 years (left panels) and ≤10 years (right panels). Discriminative performance was assessed using time-dependent ROC analysis. AUC, SE, 95% confidence CIs, and *p* values are shown in the accompanying tables. Statistical significance was defined as *p* <0.05. Across all time horizons and endpoints, the VAIL score demonstrated the highest discriminative accuracy. The diagonal line represents the reference line of no discrimination. ALBI, albumin–bilirubin; APRI, AST-to-platelet ratio index; AST, aspartate aminotransferase; CHESS-ALARM, Chronic Hepatic Event Stratification System–ALARM; EPOD, early prediction of decompensation; FIB-4, Fibrosis-4; MELD, model for end-stage liver disease; ROC, receiver operating characteristic; VAIL, VEGF-D, Angiopoietin-1, I-FABP, IL-6; VITRO, von Willebrand factor antigen-to-platelet ratio.

#### Split-sample sensitivity analysis

As a secondary analysis, the VAIL score showed consistently high discriminative performance in both the derivation and validation cohorts at short- and long-term follow-up. Discrimination was largely preserved at 3 and 10 years, whereas established clinical and fibrosis-based scores showed greater variability and attenuation in the validation cohort.

Overall, split-sample findings were concordant with bootstrap internal validation and support the robustness of the VAIL score. Detailed AUC estimates are provided in Fig. S4.

#### Impact of clinical events on survival

Kaplan-Meier analysis showed that patients who developed decompensation—either alone or in combination with PVT—had the poorest survival. Patients with HCC or isolated PVT had intermediate outcomes, with survival significantly inferior to that of individuals who remained compensated throughout follow-up (Fig. 4).

**Fig. 4:**
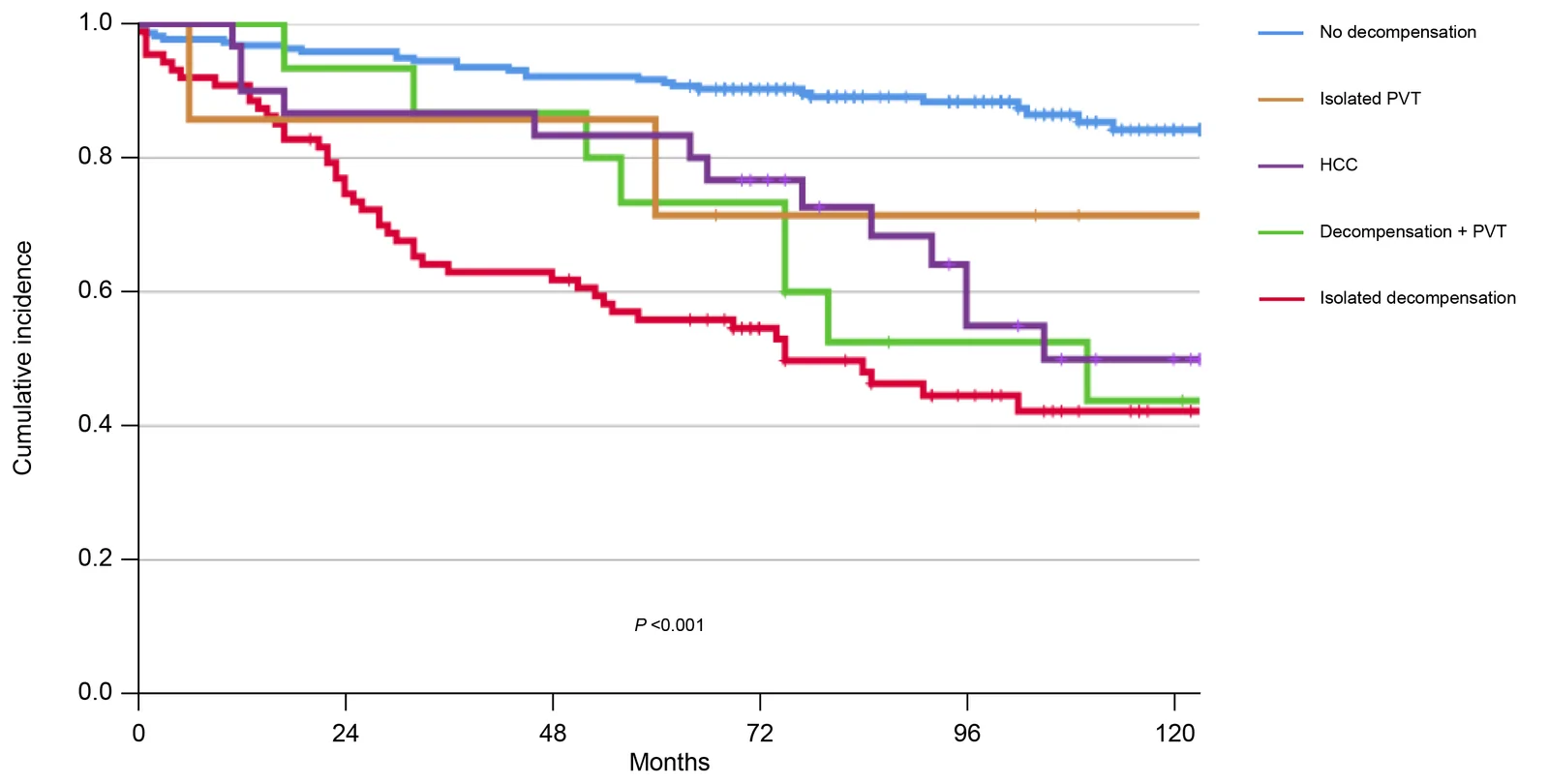
**Kaplan-Meier survival curves according to the type of the first clinical event.** Groups include the following: no event, isolated decompensation, decompensation with PVT, isolated PVT, and HCC. Censored observations for each group are shown with corresponding tick marks. Survival differed substantially across trajectories, with the poorest outcomes observed in patients who developed decompensation, either alone or with PVT, followed by those with HCC. Patients with isolated PVT had survival closer to that of patients who remained compensated. Survival was estimated using the Kaplan-Meier method and compared across groups using the log-rank test. Statistical significance was defined as *p* <0.05; the overall comparison across trajectories was significant (*p* <0.001). HCC, hepatocellular carcinoma; PVT, portal vein thrombosis.

Time-dependent Cox regression further clarified the prognostic hierarchy of major events. Hepatic decompensation was the strongest determinant of mortality, followed by HCC, whereas PVT showed no independent effect on short- or long-term survival. In the early phase, decompensation was associated with an almost 12-fold increase in mortality risk (HR 11.86), compared with a fourfold increase for HCC (HR 4.40). Over longer follow-up, hazard estimates decreased but remained substantial for both events (HR 4.5–4.9 for decompensation, with a similar magnitude for HCC). Mortality risk was highest when decompensation occurred without PVT, reinforcing that PVT does not modify prognosis and instead tends to arise as a downstream manifestation of advanced, unstable disease (Table 3).

**Table 3:** Time-dependent Cox proportional hazards regression analysis of the impact of major clinical events on all-cause mortality.

Time	Estimation		SE		*p* value		95% CI	
	β	HR	SE (β)	SE (HR)	*p* (β)	*p* (HR)	95% CI (β)	95% CI (HR)
**Short term (3 years)**								
Decompensation	2.474	11.867	0.376	4.458	<0.001	<0.001	1.737–3.210	5.683–24.783
PVT	-0.579	0.560	0.755	0.423	0.443	0.443	-2.059 to 0.902	0.127–2.463
HCC	1.498	4.475	0.395	1.770	<0.001	<0.001	0.723–2.274	2.061–9.715

**Long term (10 years)**								
Decompensation	1.505	4.505	0.215	0.969	<0.001	<0.001	1.083–1.927	2.955–6.869
PVT	0.220	1.246	0.367	0.457	0.548	0.548	-0.499 to 0.939	0.607–2.558
HCC	1.603	4.967	0.224	1.113	<0.001	<0.001	1.164–2.042	3.202–7.706

Time-dependent Cox proportional hazards models were used to evaluate the association between incident hepatic decompensation, PVT, and HCC with all-cause mortality during short-term (3 years) and long-term (10 years) follow-up. Clinical events were modelled as time-varying covariates that dynamically contributed to mortality risk from the time of their occurrence. HRs with 95% CIs are reported. Values of *p* were obtained using Wald tests. Statistical significance was defined as *p* <0.05. Models were adjusted for age, sex, and baseline MELD score. Time was structured using a counting process (start–stop) approach. Decompensation and HCC were significant predictors of mortality at both horizons, whereas PVT had no measurable effect on survival. Reference categories consisted of patients without the corresponding event. β, coefficient; HCC, hepatocellular carcinoma; HR, hazard ratio; MELD, model for end-stage liver disease; PVT, portal vein thrombosis.

The Sankey diagram further displays cause-specific mortality according to preceding clinical trajectory, with terminal nodes indicating liver-related death, HCC-related death, or death occurring as the first recorded event; flow thickness is proportional to the relative frequency of each pathway (Fig. 5). Three key patterns emerge: (1) decompensation is by far the earliest and most frequent transition; (2) it constitutes the dominant pathway leading to liver-related death; and (3) PVT contributes minimally to the overall prognostic landscape, consistent with its behaviour as a secondary, downstream event rather than an independent driver of disease progression.

**Fig. 5:**
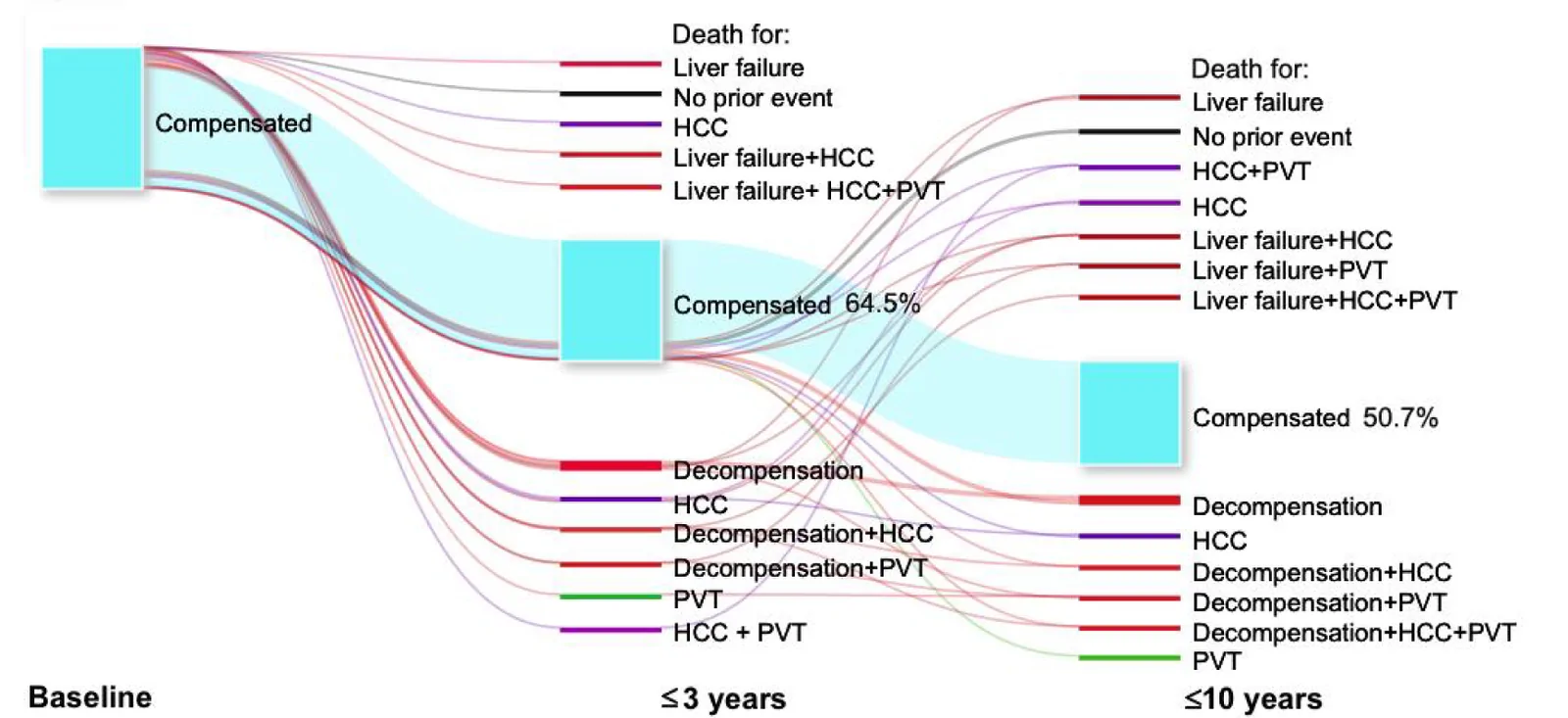
**Sankey diagram of clinical trajectories from compensated status to advanced disease states and cause-specific mortality.** The diagram displays transitions from compensated status at baseline to clinical outcomes occurring within 3 and 10 years in patients with cACLD. Events are ordered by frequency. Terminal mortality nodes indicate cause-specific death, including liver-related death, HCC-related death, and death occurring as the first recorded event. The thickness of each flow is proportional to the relative frequency of the corresponding clinical trajectory. No formal statistical test was applied. The most common outcomes were hepatic decompensation, HCC, and their combinations, followed by PVT and death. cACLD, compensated advanced chronic liver disease; HCC, hepatocellular carcinoma; PVT, portal vein thrombosis.

## Discussion

In this prospective cohort of patients with cACLD, we demonstrate that hepatic decompensation is substantially more frequent, occurs earlier, and carries far greater prognostic weight than PVT. These findings delineate distinct clinical trajectories and molecular signatures of decompensation and PVT, challenging the traditional view that PVT is the primary driver of clinical deterioration in cACLD.^1^^,^^4^

Decompensation emerged as the pivotal inflection point in the natural history of cACLD. In most patients who developed both events, decompensation preceded PVT, suggesting that progressive hepatic and systemic dysfunction creates a prothrombotic milieu through inflammation, endothelial injury, gut barrier disruption, and microvascular dysfunction. Therefore, PVT appears to represent a downstream manifestation of disease progression rather than an independent precipitant.^3^^,^[19], [20], [21], [22] These findings should not be interpreted as excluding a potential contribution of PVT to subsequent clinical deterioration in selected patients, particularly in more advanced disease stages or in the setting of extensive/occlusive thrombosis. However, in this rigorously compensated cohort, incident non-malignant PVT did not behave as an independent aggravating factor *per se*, but rather as a marker of increasing severity of CLD, PH, and systemic deterioration.^6^^,^^7^ This interpretation is supported by the restricted temporal window in which PVT occurred, its low incidence as a first event, and the time-dependent survival analyses across the full observation period, in which decompensation remained the dominant prognostic event, whereas PVT showed limited independent association with mortality. These findings support a reassessment of the clinical weight attributed to incident non-malignant PVT in risk stratification and management decisions in cACLD.

Baseline MELD further distinguished these trajectories. Patients who developed decompensation had higher MELD values, consistent with previous studies linking MELD to the risk of first or recurrent decompensation.^23^^,^^24^ In contrast, patients with isolated PVT had MELD values comparable to those of patients who remained compensated, suggesting that PVT may arise from localized vascular or hypoxic stress rather than global hepatic failure.

Consistent with a recent meta-analysis,^25^ albumin was the strongest short-term predictor of decompensation, whereas both albumin and INR predicted long-term risk. This pattern is biologically plausible, as INR has a limited dynamic range and is less sensitive to early pathophysiological changes.

Comprehensive proteomic profiling revealed marked biological divergence between decompensation and PVT. Although isolated PVT was associated with a narrow signature, primarily Tie2 and mesothelin, decompensation was characterized by broad multisystem dysregulation, encompassing angiogenesis (Angpt1↓, Angpt2↑, and VEGF-D↑), inflammation (IL-6↑, IL-8↑, and TNF-α↑), matrix remodelling (HA↑, MMP-7↑, and MMP-9↑), endothelial activation (Nectin-2↑ and Endoglin↑), and gut barrier disruption (I-FABP↑). This extensive upstream signature supports the concept of decompensation as a systemic process driven by sustained PH, chronic inflammation, and microvascular dysfunction.^6^^,^^26^^,^^27^

Building on these biological insights, we derived the VAIL score, a mechanistically grounded prognostic tool integrating vascular (VEGF-D), angiogenic (Angiopoietin-1), inflammatory (IL-6), and intestinal injury (I-FABP) pathways. By capturing multisystem biological instability preceding clinical decompensation, VAIL consistently outperformed established clinical and fibrosis-based scores at both short- and long-term horizons, including in patients who later developed PVT.^6^^,^^27^ Bootstrap validation confirmed the robustness and generalizability of the VAIL score, which retained statistical significance and effect size across 1,000 resampled datasets. Although the VAIL score showed consistent performance across bootstrap resampling and split-sample sensitivity analyses, it should be regarded as a candidate biomarker-based risk stratification tool rather than a model ready for routine clinical implementation. External validation in independent, preferably multicentre cohorts is required before its use can be recommended in clinical practice. The lack of such validation reflects the difficulty of identifying comparable cohorts with rigorously defined compensated disease, long-term follow-up, standardized HVPG/LSM/endoscopic characterization, and stored biological samples suitable for parallel biomarker assessment. In contrast, associations for established clinical and fibrosis-based scores attenuated after resampling, suggesting limited reproducibility. Although biomarker-based scores such as VITRO, which combines von Willebrand factor and platelet count, have shown prognostic value in cirrhosis,[28], [29], [30] VITRO did not outperform clinical scores in our cohort and was clearly inferior to VAIL. This likely reflects the broader mechanistic coverage of VAIL, integrating complementary biological pathways rather than a single vascular axis.

Together, these findings support a refined conceptual framework in which decompensation represents the primary driver of morbidity and mortality in cACLD, whereas PVT reflects a secondary, biologically distinct process with limited prognostic influence. Incorporating biomarker profiling into clinical assessment may enable earlier identification of high-risk patients and facilitate more targeted preventive strategies.

The strengths of this study include its prospective design, long-term follow-up, comprehensive baseline characterization (HVPG, LSM, endoscopy, and deep biomarker profiling), and the availability of serial biomarker measurements. The single-centre design ensured homogeneous assessment, follow-up, and biomarker handling but may limit generalizability across centres with different aetiologic case-mix, surveillance intensity, therapeutic practices, and laboratory platforms. Accordingly, the calibration and clinical applicability of the VAIL score should be confirmed in external cohorts before it is used for patient-level decision-making. Additional limitations include the single-centre design, observational analyses, and the absence of systematic assessment of therapies such as nonselective β-blockers or anticoagulation. Treatment exposure during follow-up should also be considered when interpreting event rates. Follow-up information on the main treatments of interest was available; however, nonselective β-blockers, aetiologic therapies, and anticoagulation were administered according to clinical indications and evolving standards of care rather than by protocol. Exploratory association analyses confirmed this indication-driven pattern, with β-blocker use strongly associated with baseline large varices and subsequent decompensation, and enoxaparin use associated with more advanced or complicated clinical trajectories. The independent causal impact of these treatments on disease course could therefore not be reliably disentangled from the underlying severity of PH and disease progression. Importantly, the VAIL score was derived from biomarkers measured at enrolment, before subsequent treatment decisions, and therefore reflects baseline biological risk rather than treatment response. Moreover, anticoagulation was used therapeutically after incident PVT, not prophylactically, and is therefore unlikely to explain the occurrence of PVT as a first event. Similarly, Direct-Acting Antiviral (DAA) therapy became available during the study period and was initially restricted by regulatory criteria, limiting its potential influence on early follow-up events in patients with fully compensated disease. Nevertheless, residual treatment-related confounding or event modification cannot be fully excluded. Exploratory association analyses of follow-up treatments confirmed an indication-driven pattern of treatment allocation, particularly for nonselective β-blockers and enoxaparin, supporting the decision not to model these dynamic exposures as independent causal predictors of disease progression. Finally, although the four VAIL biomarkers are measurable using ELISA-based platforms and are therefore technically feasible, assay standardization, inter-laboratory reproducibility, platform comparability, and clinically validated thresholds will be needed before translation into routine care. Future studies should validate these findings in independent cohorts and examine whether biomarker-guided interventions can prevent decompensation or modify disease trajectory.^31^

In conclusion, hepatic decompensation is the earliest and most prognostically meaningful event in cACLD, whereas PVT arises later and reflects a distinct, less impactful biological process. Recognition of their divergent temporal and molecular profiles provides a framework for refined risk stratification and supports the rationale for biomarker-informed preventive strategies in advanced CLD.

## Abbreviations

ALBI, albumin–bilirubin; ALT, alanine aminotransferase; ANGPTL4, Angiopoietin-like 4; Ang-1, Angiopoietin-1; Ang-2, Angiopoietin-2; APRI, AST-to-platelet ratio index; AST, aspartate aminotransferase; AVEN, Area Vasta Emilia Nord; CA9, carbonic anhydrase 9; CHESS-ALARM, Chronic Hepatic Event Stratification System–ALARM; cACLD, compensated advanced chronic liver disease; CLD, chronic liver disease; CRP, C-reactive protein; EPOD, early prediction of decompensation; FDR, false discovery rate; FIB-4, Fibrosis-4; HA, hyaluronic acid; HCC, hepatocellular carcinoma; HR, hazard ratio; HVPG, hepatic venous pressure gradient; I-FABP, Intestinal fatty acid-binding protein; INR, international normalized ratio; LASSO, least absolute shrinkage and selection operator; LSM, liver stiffness measurement; MELD, model for end-stage liver disease; MMP, matrix metalloproteinase; ROC, receiver operating characteristic; PH, portal hypertension; PVT, portal vein thrombosis; TGF-β1, transforming growth factor beta-1; Tie2, tyrosine kinase with immunoglobulin-like and EGF-like domains 2; TNF-α, tumour necrosis factor alpha; TPO, thrombopoietin; VAIL, VEGF-D, Angiopoietin-1, I-FABP, IL-6; VEGF, Vascular endothelial growth factor; VEGF-D, vascular endothelial growth factor D; VITRO, von Willebrand factor antigen-to-platelet ratio.

## Authors’ contributions

Conceptualization: EV, GG. Formal analysis: EV, RD, RMC. Data curation: RMC, ES, FM. Methodology: EV, ES, FD. Investigation: RMC, FM. Software: FS. Writing—original draft: EV, RD, FD. Writing—review & editing: all authors. Funding acquisition: EV, GG. Supervision: EV, GG.

## Data availability

All clinical and biological variables are available on reasonable request for academic researchers in hepatology research (contact: erica.villa@irccsdebellis.it).

## Financial support

This study was supported by 10.13039/501100007076Fondazione Italiana per la Ricerca sul Cancro (AIRC) (grant no. IG 2020-ID, 24858 to EV) and by Ministero della Salute, Ricerca Corrente 2025 (to GG).

The funders had no role in the design, conduct, analysis, interpretation, or reporting of this study.

## Conflicts of interest

The authors declare no conflicts of interest.

Please refer to the accompanying ICMJE disclosure forms for further details.

## References

[1] Stine J.G., Shah P.M., Cornella S.L. (2015). Portal vein thrombosis, mortality and hepatic decompensation in patients with cirrhosis: a meta-analysis. World J Hepatol.

[2] Senzolo M., Garcia-Tsao G., García-Pagán J.C. (2021). Current knowledge and management of portal vein thrombosis in cirrhosis. J Hepatol.

[3] Nery F., Chevret S., Condat B. (2015). Causes and consequences of portal vein thrombosis in 1,243 patients with cirrhosis: results of a longitudinal study. Hepatology.

[4] Xian J., Tang Y., Shao H. (2021). Effect of portal vein thrombosis on the prognosis of patients with cirrhosis without a liver transplant: a systematic review and meta-analysis. Medicine (Baltimore).

[5] Anton A., Campreciós G., Pérez-Campuzano V. (2022). The pathophysiology of portal vein thrombosis in cirrhosis: getting deeper into Virchow's triad. J Clin Med.

[6] Engelmann C., Clària J., Szabo G. (2021). Pathophysiology of decompensated cirrhosis: portal hypertension, circulatory dysfunction, inflammation, metabolism and mitochondrial dysfunction. J Hepatol.

[7] D’Amico G., Garcia-Tsao G., Pagliaro L. (2006). Natural history and prognostic indicators of survival in cirrhosis: a systematic review of 118 studies. J Hepatol.

[8] D’Amico G., Morabito A., D’Amico M. (2018). Clinical states of cirrhosis and competing risks. J Hepatol.

[9] De Franchis R. (2015). Expanding consensus in portal hypertension: report of the Baveno VI Consensus Workshop: stratifying risk and individualizing care for portal hypertension. J Hepatol.

[10] Pugh R.N., Murray-Lyon I.M., Dawson J.L. (1973). Transection of the oesophagus for bleeding oesophageal varices. Br J Surg.

[11] Kamath P.S., Wiesner R.H., Malinchoc M. (2001). A model to predict survival in patients with end-stage liver disease. Hepatology.

[12] Schneider A.R.P., Schneider C.V., Schneider K.M. (2022). Early prediction of decompensation (EPOD) score: non-invasive determination of cirrhosis decompensation risk. Liver Int.

[13] Wai C.T., Greenson J.K., Fontana R.J. (2003). A simple noninvasive index can predict both significant fibrosis and cirrhosis in patients with chronic hepatitis C. Hepatology.

[14] Johnson P.J., Berhane S., Kagebayashi C. (2015). Assessment of liver function in patients with hepatocellular carcinoma: a new evidence-based approach—the ALBI grade. J Clin Oncol.

[15] Sterling R.K., Lissen E., Clumeck N. (2006). Development of a simple noninvasive index to predict significant fibrosis in patients with HIV/HCV coinfection. Hepatology.

[16] Schwarzer R., Reiberger T., Mandorfer M. (2020). The von Willebrand factor antigen to platelet ratio (VITRO) score predicts hepatic decompensation and mortality in cirrhosis. J Gastroenterol.

[17] Wong Y.J., Li J., Liu C. (2022). CHESS-ALARM score to stratify decompensation risk in compensated advanced chronic liver disease patients: an international multicenter study. J Gastroenterol Hepatol.

[18] European Association for the Study of the Liver (2018). EASL Clinical Practice Guidelines: management of hepatocellular carcinoma. J Hepatol.

[19] Giannini E.G., Stravitz R.T., Caldwell S.H. (2016). Portal vein thrombosis and chronic liver disease progression: the closer you look the more you see. Hepatology.

[20] Chen Z., Ran T., Cao H. (2021). The impact of portal vein thrombosis on the prognosis of patients with cirrhosis: a retrospective propensity-score matched study. Front Med (Lausanne).

[21] Noronha Ferreira C., Marinho R.T., Cortez-Pinto H. (2019). Incidence, predictive factors and clinical significance of development of portal vein thrombosis in cirrhosis: a prospective study. Liver Int.

[22] Kondili L.A., Zanetto A., Quaranta M.G. (2024). Predicting de-novo portal vein thrombosis after HCV eradication: a long-term competing risk analysis in the ongoing PITER cohort. United Eur Gastroenterol J.

[23] Dultz G., Seelhof M., Herrmann E. (2013). Baseline MELD score predicts hepatic decompensation during antiviral therapy in patients with chronic hepatitis C and advanced cirrhosis. PLoS One.

[24] Balcar L., Tonon M., Semmler G. (2022). Risk of further decompensation/mortality in patients with cirrhosis and ascites as the first single decompensation event. JHEP Rep.

[25] Gananandan K., Singh R., Mehta G. (2024). Systematic review and meta-analysis of biomarkers predicting decompensation in patients with compensated cirrhosis. BMJ Open Gastroenterol.

[26] Simonetto D.A., Yang H., Yin M. (2015). Chronic passive venous congestion drives hepatic fibrogenesis via sinusoidal thrombosis and mechanical forces. Hepatology.

[27] Trebicka J., Fernandez J., Papp M. (2021). PREDICT identifies precipitating events associated with the clinical course of acutely decompensated cirrhosis. J Hepatol.

[28] Ferlitsch M., Reiberger T., Hoke M. (2012). von Willebrand factor as new noninvasive predictor of portal hypertension, decompensation and mortality in patients with liver cirrhosis. Hepatology.

[29] Mandorfer M., Schwabl P., Paternostro R. (2018). Von Willebrand factor indicates bacterial translocation, inflammation, and procoagulant imbalance and predicts complications independently of portal hypertension severity. Aliment Pharmacol Ther.

[30] Simbrunner B., Villesen I.F., Scheiner B. (2023). Von Willebrand factor processing in patients with advanced chronic liver disease and its relation to portal hypertension and clinical outcome. Hepatol Int.

[31] Trebicka J., Hernaez R., Shawcross D.L. (2024). Recent advances in the prevention and treatment of decompensated cirrhosis and acute-on-chronic liver failure (ACLF) and the role of biomarkers. Gut.

