# Supplementary material for: First decompensation outweighs portal vein thrombosis in cACLD: Molecular profiling and derivation of the VAIL score
Source: JHEP Rep. 2026 Aug 29;8(11):102017. doi: 10.1016/j.jhepr.2026.102017 (PMC13673353; doi:10.1016/j.jhepr.2026.102017)
Supplement: Supplementary file 1 [file mmc1.pdf]

First decompensation outweighs portal vein thrombosis in cACLD: Molecular profiling and derivation of the VAIL score

Erica Villa, Rosina Maria Critelli, Rossella Donghia, Endrit Sahini, Fabiola Milosa, Filippo Scianò, Francesco Dituri, Gianluigi Giannelli

Table of contents

Supplementary methods……………………………………………………………2
Supplementary figures…………………………………………………………….....3
Supplementary tables……………………………………………………………....8

Supplementary methods

Quantification of serum biomarkers

Serum levels of Angiopoietin-1 (Angpt1), Angiopoietin-2 (Angpt2), Tie2,Vascular Endothelial Growth Factors (VEGFA, VEGFD), Angiopoietin-like 4 (ANGPTL4), Angiogenin, C-Reactive Protein (CRP), Adiponectin, Mesothelin, E-Cadherin, SPINK1, Endoglin, Matrix Metalloproteinase 7 (MMP-7), Nectin 2, Surfactant Protein D (SP-D), Serpin A1, Tissue Inhibitor of Metalloproteinases 1 (TIMP-1), Interleukins (IL1, IL10, IL6, IL8), Cartilage Oligomeric Matrix Protein (COMP), Matrix Metalloproteinase 9 (MMP 9), Osteopontin (OPN), Vascular Endothelial Growth Factor Receptor 2 (VEGF R2), CD14, Transforming Growth Factor beta 1 (TGF-β1), TNF-α, I-FABP, and Carbonic Anhydrase 9 (CA9) were quantified by using the Ella System (Biotechne, San Jose, CA, USA),following provided instructions. The system is fully automated and the quantity of the analyte is directly calculated by the software through the interpolation of a certified calibration curve associated with the loaded cartridge.

Briefly, serum samples were diluted with sample diluent according to the suggested indications and this solution was added to each sample wells on the ELLA cartridge. Wash buffer was added into all buffer inlets on the ELLA cartridge. The cartridge was then loaded into the ELLA instrument (Biotechne) for analysis. Serum samples were run in triplicate and results were calculated by the Simple Plex Explorer software (Biotechne) and data are expressed as pg/mL or ng/mL.

Hyaluronic acid (HA) levels were determined by ELISA assay (FineTest®, Wuhan Fine Biotech Co., Ltd. Wuhan, Hubei, China) according to the manufacturer's indications. Analyses were assayed in duplicate and read on the Victor 3 V microplate reader at 450 nm (PerkinElmer, Waltham, MA, United States). Data are expressed as ng/mL.

The serum level of ADMA (asymmetric dimethylarginine) was measured using the Human ADMA ELISA Kit (Cusabio, Wuhan, China), which has a detection range of 7.8 ng/mL to 500 ng/mL. All assay procedures were performed according to the manufacturer’s instructions. The absorbances of standards and samples were measured at 450 nm using a plate reader GloMax®-Multi Microplate Multimode Reader (Promega Corporation, Madison, WI, USA). Data are expressed as ng/mL. Thrombopoietin (TPO) and Von Willebrand Factor (vWF) concentrations were measured using multiplex fluorescent microparticle-based immunoassays (Human Magnetic Luminex Assay, R&D Systems, Minneapolis, MN) according to the manufacturer's specifications. Plates were read immediately on the FLEX MAP 3D instrument (Bio-Rad, Hercules, CA) and raw data were analyzed with the Bio Plex 6.2 software (Bio-Rad) to obtain concentration values. Data are expressed as pg/mL.

Supplementary figures

Fig. S1 – Flowchart of the study

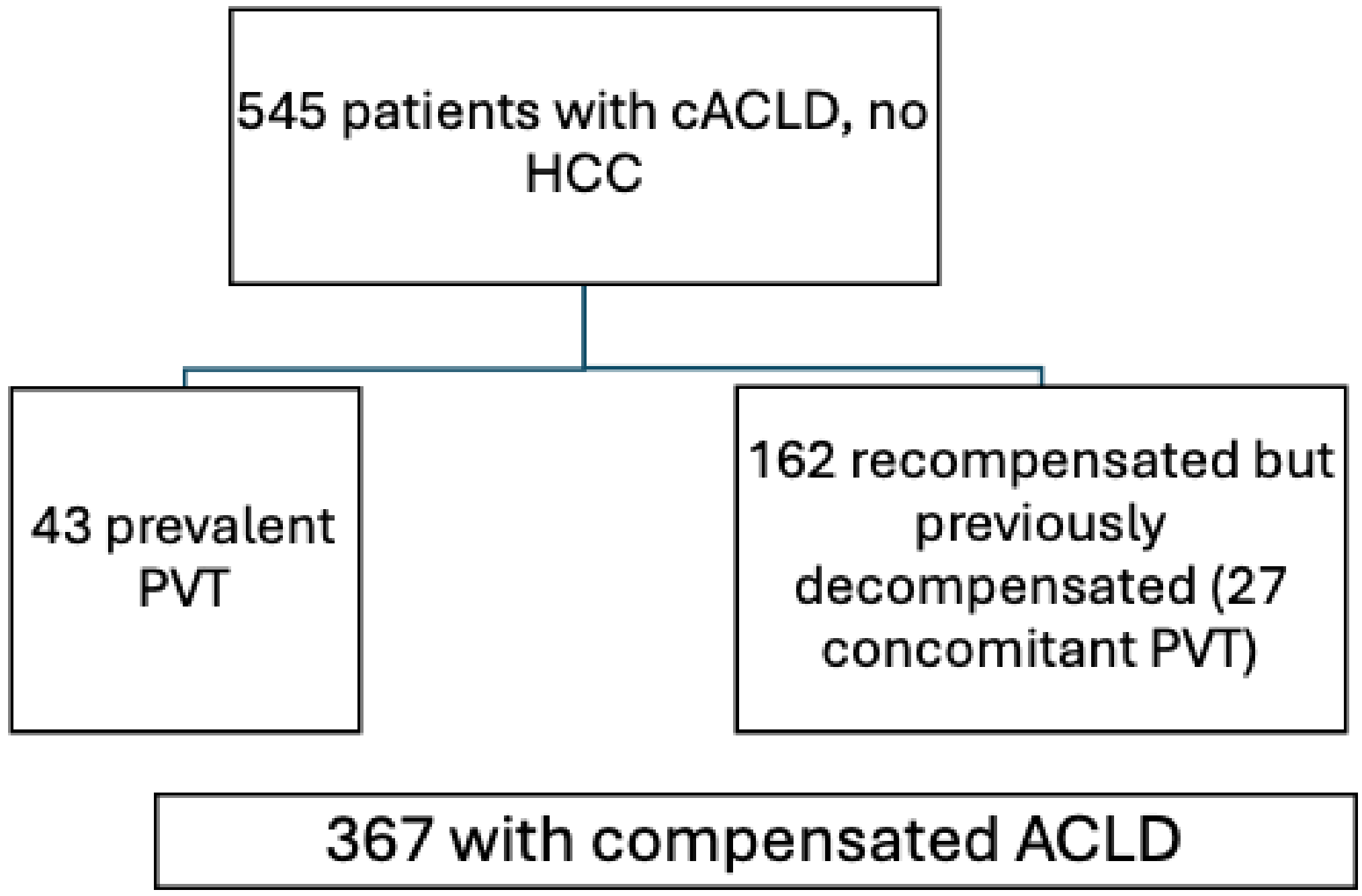

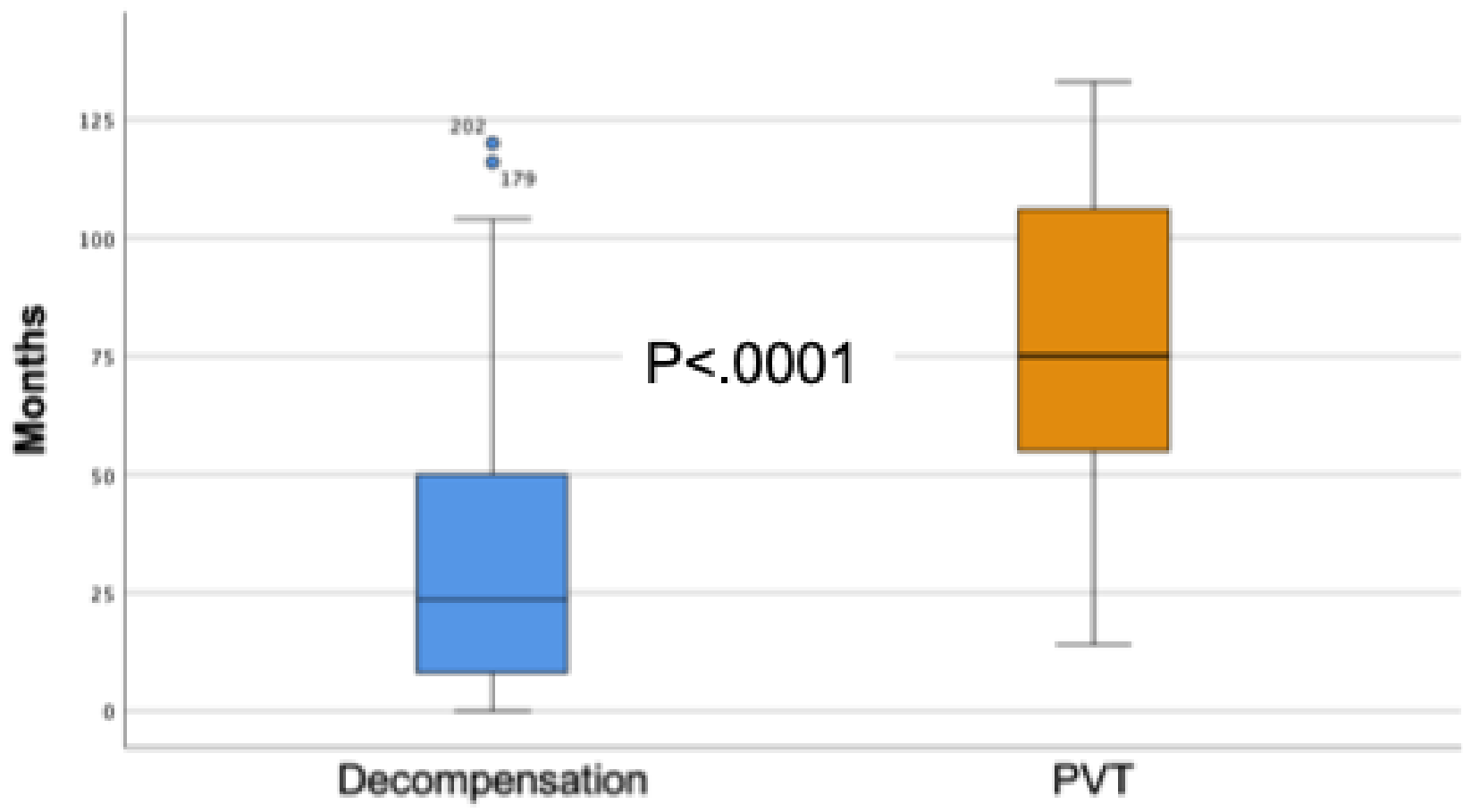

Fig. S2. Boxplots showing the distribution of time to first event among patients who developed isolated hepatic decompensation or portal vein thrombosis (PVT) associated with decompensation. Boxes represent the interquartile range (IQR), horizontal lines indicate the median, whiskers extend to 1.5×IQR, and dots denote outliers (Mann–Whitney U test).

Decompensation occurred earlier and exhibited greater temporal variability, whereas PVT presented later and within a narrower time window.

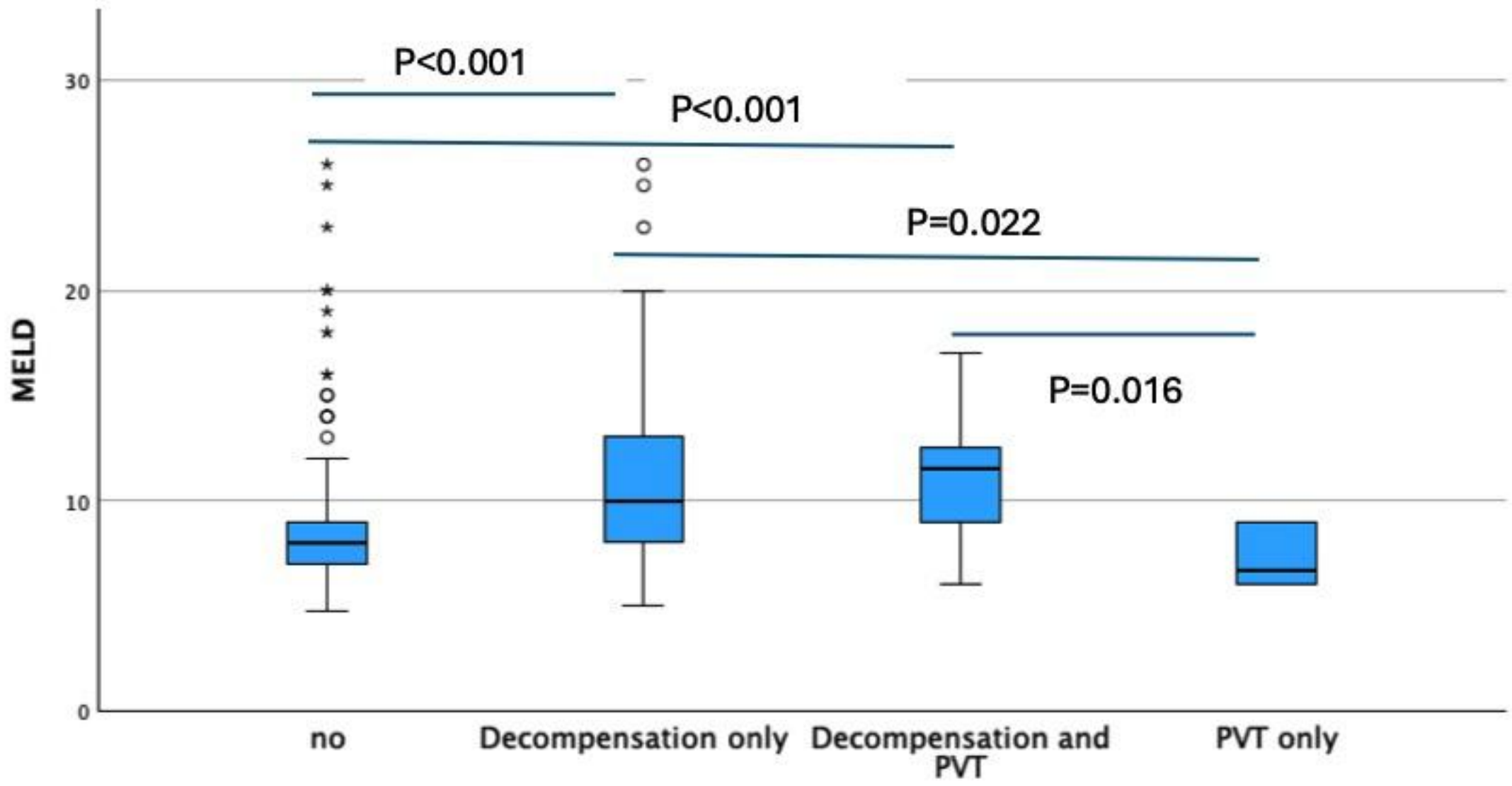

Fig. S3. Baseline MELD scores across patient subgroups stratified by clinical outcomes - Box plots show Model for End-Stage Liver Disease (MELD) scores at enrollment in patients who remained free of decompensation ("no"), developed decompensation only, both decompensation and portal vein thrombosis (PVT), or PVT only. MELD scores were significantly higher in patients who developed decompensation - with or without PVT - compared to those who remained compensated or developed PVT alone ($p<0.001$, Kruskal–Wallis test. Pairwise comparisons are shown in the figure; statistical significance was defined as $p<0.05$). These results highlight the association between more advanced liver dysfunction and the risk of decompensation.

≤3 years **≤10 years**

Decompensation alone

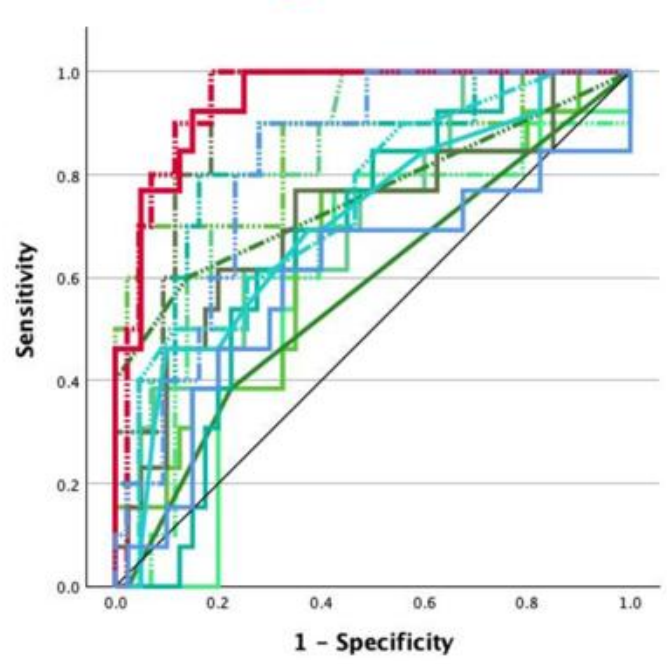

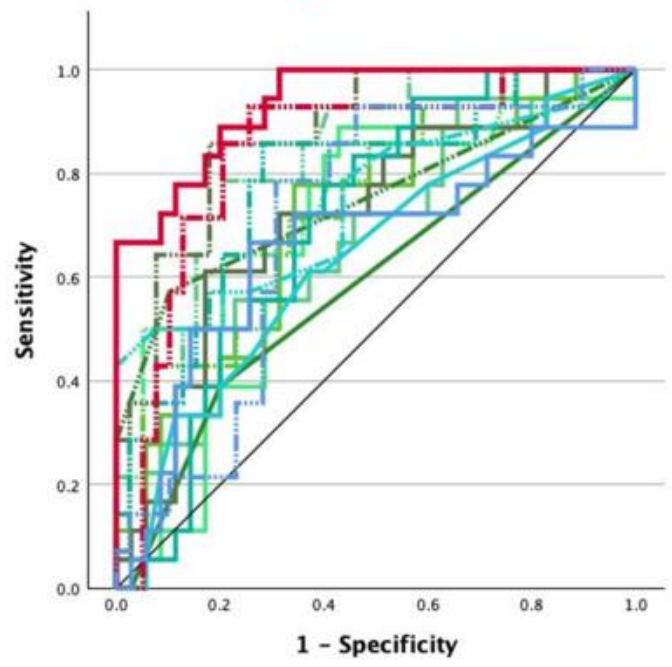

Scores	Groups	AUC	Se (AUC)	p	95% CI		
ALBI	D	.70	.08	.010	.55	to	.86
	V	.84	.05	<0.001	.72	to	.95
APRI	D	.60	.08	.217	.43	to	.77
	V	.65	.10	.144	.44	to	.85
CHESS-ALARM	D	.65	.08	.078	.48	to	.82
	V	.84	.08	.000	.68	to	1.00
CHILD-PUGH	D	.57	.09	.425	.39	to	.75
	V	.75	.10	.011	.55	to	.95
EPOD	D	.71	.08	.017	.53	to	.88
	V	.89	.03	<0.001	.83	to	.98
FIB-4	D	.67	.07	.022	.52	to	.82
	V	.84	.07	<0.001	.70	to	.98
MELD	D	.70	.08	.013	.54	to	.87
	V	.74	.08	.005	.57	to	.90
VITRO	D	.59	.10	.362	.39	to	.79
	V	.82	.06	<0.001	.70	to	.94
VAIL	D	.94	.03	<0.001	.88	to	1.00
	V	.94	.03	<0.001	.87	to	1.00

Scores	Groups	AUC	Se (AUC)	p	95% CI		
ALBI	D	.67	.07	.022	.52	to	.82
	V	.83	.05	<0.001	.72	to	.95
APRI	D	.67	.07	.021	.52	to	.83
	V	.76	.07	.001	.61	to	.91
CHESS-ALARM	D	.70	.07	.006	.56	to	.85
	V	.74	.07	.001	.59	to	.88
CHILD-PUGH	D	.58	.08	.294	.42	to	.75
	V	.74	.08	.005	.57	to	.92
EPOD	D	.71	.07	.003	.57	to	.86
	V	.82	.04	<0.001	.78	to	.97
FIB-4	D	.70	.07	.004	.56	to	.84
	V	.74	.07	<0.001	.64	to	.92
MELD	D	.63	.08	.089	.47	to	.79
	V	.74	.08	.005	.57	to	.91
VITRO	D	.65	.08	.070	.48	to	.82
	V	.70	.07	.008	.55	to	.85
VAIL	D	.93	.03	<0.001	.87	to	.99
	V	.84	.06	<0.001	.71	to	.96

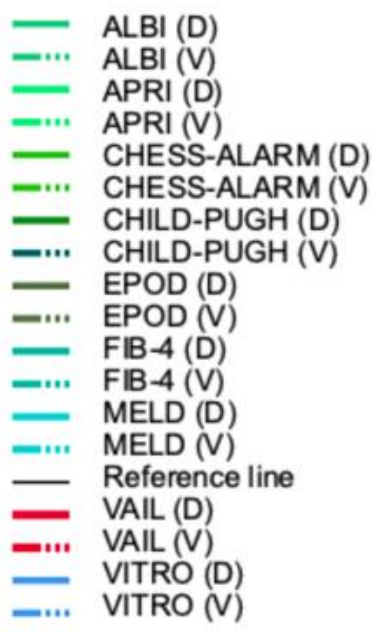

Decompensation + PVT

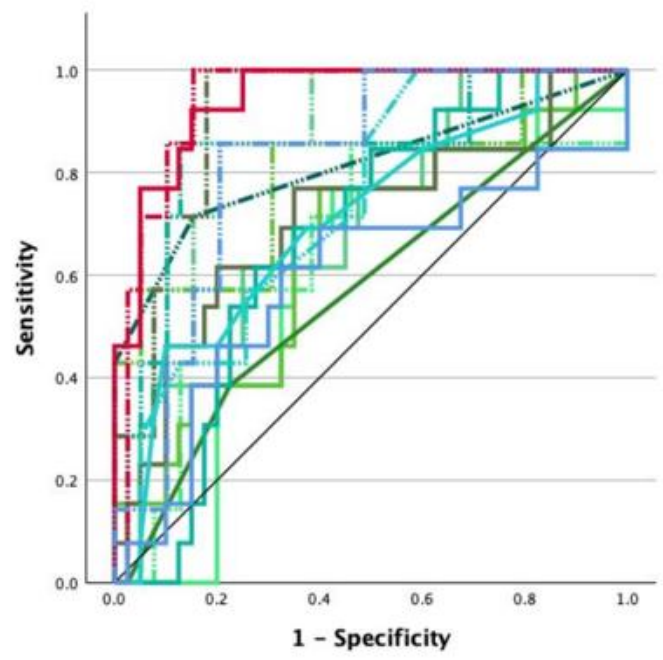

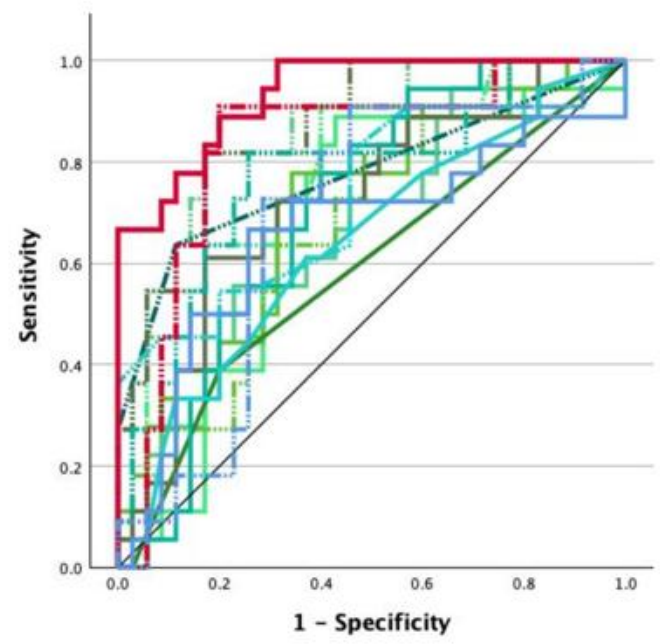

Scores	Groups	AUC	Se (AUC)	p	95% CI		
ALBI	D	.70	.08	.010	.55	to	.86
	V	.86	.06	<0.001	.75	to	.98
APRI	D	.60	.08	.217	.43	to	.77
	V	.66	.12	.177	.42	to	.90
CHESS-ALARM	D	.65	.08	.078	.48	to	.82
	V	.79	.10	.007	.58	to	1.00
CHILD-PUGH	D	.57	.09	.425	.39	to	.75
	V	.81	.11	.004	.59	to	1.02
EPOD	D	.71	.08	.017	.53	to	.88
	V	.91	.04	<0.001	.82	to	.99
FIB-4	D	.67	.07	.022	.52	to	.82
	V	.84	.09	.000	.66	to	1.02
MELD	D	.70	.08	.013	.54	to	.87
	V	.73	.08	.008	.56	to	.90
VITRO	D	.59	.10	.362	.39	to	.79
	V	.82	.07	<0.001	.68	to	.95
VAIL	D	.94	.03	<0.001	.88	to	1.00
	V	.94	.03	<0.001	.87	to	1.01

Scores	Groups	AUC	Se (AUC)	p	95% CI		
ALBI	D	.67	.07	.022	.52	to	.82
	V	.82	.06	<0.001	.74	to	.98
APRI	D	.67	.07	.021	.52	to	.83
	V	.79	.07	<0.001	.64	to	.94
CHESS-ALARM	D	.70	.07	.006	.56	to	.85
	V	.68	.08	.034	.51	to	.85
CHILD-PUGH	D	.58	.08	.294	.42	to	.75
	V	.77	.09	.003	.59	to	.96
EPOD	D	.71	.07	.003	.57	to	.86
	V	.82	.05	<0.001	.75	to	.97
FIB-4	D	.70	.07	.004	.56	to	.84
	V	.77	.08	.001	.60	to	.93
MELD	D	.63	.08	.089	.47	to	.79
	V	.73	.09	.016	.54	to	.91
VITRO	D	.65	.08	.070	.48	to	.82
	V	.68	.08	.035	.51	to	.85
VAIL	D	.93	.03	<0.001	.87	to	.99
	V	.83	.07	<0.001	.69	to	.97

Fig. S4 - Split-sample sensitivity analysis of the VAIL score.

Time-dependent receiver operating characteristic (ROC) curves evaluating the discriminative performance of the VAIL score and established prognostic indices for

incident hepatic decompensation (with or without portal vein thrombosis) at ≤3-year and ≤10-year follow-up.

The full cohort was randomly divided into a derivation cohort (n = 200) and a validation cohort (n = 167), drawn from the same prospective population. ROC curves are shown separately for the derivation and validation cohorts as an internal split-sample sensitivity analysis to assess the stability of discrimination across independent subsamples.

Area under the curve (AUC), standard error, values with 95% confidence intervals and p values are reported in the accompanying tables. Statistical significance was defined as $p<0.05$.

(D: derivation; V: Validation)

Supplementary tables

Table S1 - Anatomical, occlusive, and follow-up characteristics of incident non-malignant portal vein thrombosis. PVT characteristics were assessed in 22 patients with incident non-malignant PVT confirmed by contrast-enhanced CT or MRI. Thrombi were classified according to main portal vein involvement, degree of luminal occlusion, extension within the porto-mesenteric axis, presence of cavernous transformation, progression on follow-up imaging, and response to anticoagulation. Complete main portal vein occlusion was defined as absence of residual lumen in the main portal vein. Occlusion categories are mutually exclusive, whereas anatomical extension categories, including superior mesenteric vein and splenic vein/confluence involvement, are not mutually exclusive. All patients with incident PVT received anticoagulation; recanalization was categorized as complete, partial, or absent on follow-up imaging. Percentages are calculated using the total number of incident PVT cases as denominator. MPV, main portal vein; PVT, portal vein thrombosis; SMV, superior mesenteric vein. Data are descriptive; no formal statistical test was applied.

**PVT feature**	**n (%)**
Main portal vein involved	18 (81.8)
Complete main portal vein occlusion	2 (9.1)
>50% but incomplete MPV occlusion	12 (54.5)
<50% MPV occlusion / branch-only PVT	8 (36.4)
SMV involvement	10 (45.5)
Splenic vein / confluence involvement	9 (40.9)
Cavernous transformation	1 (4.5)
Progression on follow-up	6 (27.3)
Recanalization after anticoagulation	
Complete	2 (9.1)
Partial	10 (45.5)
No recanalization	10 (45.5)

Table S2 - Internal validation of multivariable models for prediction of hepatic decompensation. Internal validation of multivariable Cox proportional hazards models was performed using bootstrap resampling (1,000 iterations). In each iteration, the model was refitted in a bootstrap sample and applied to the original cohort to estimate optimism-corrected hazard ratios (HRs), 95% confidence intervals (CIs), and P values. Reported coefficients represent the average estimates across all bootstrap samples. The VAIL score remained the only independent and statistically significant predictor of incident decompensation after resampling, with minimal coefficient shrinkage and preserved effect size. In contrast, estimates for APRI, Child–Pugh, EPOD, FIB-4, MELD, ALBI, and VITRO were attenuated and no longer significant, indicating limited reproducibility. This procedure was used to assess model stability and to quantify potential overfitting. p values were obtained using Wald tests. Statistical significance was defined as p<0.05.

Decompensation alone (≤ 3 years)								
	Variables in the Equation					Bootstrap ˆ		
**Scores**	β	Exp (β)	se (β)	p	95% C.I. for Exp (β)	β	se (β)	95% C.I. for Exp (β)
ALBI	1.96	7.12	.35	0.21	0.31 –16.28	1.96	2.88	–3.19 to 5.96
APRI	–0.06	0.93	1.58	0.87	0.42–2.06	–0.06	0.80	–1.63 to 1.17
CHESS-ALARM	0.51	1.67	.40	0.15	0.82–3.38	0.51	0.59	–0.66 to 1.31
CHILD-PUGH	0.96	2.63	.40	0.01	1.18–5.87	0.96	1.62	–1.01 to 2.54
EPOD	–0.55	0.57	.56	0.32	0.19–1.72	–0.58	1.12	–2.15 to 1.94
FIB-4	–0.15	0.86	.17	0.37	0.61–1.20	–0.15	0.42	–0.79 to 0.42
MELD	0.01	1.00	.09	0.97	0.84–1.19	0.00	0.23	–0.33 to 0.36
VITRO	0.01	1.00	.001	0.16	0.99–1.02	0.00	0.09	–0.08 to 0.69

VAIL	1.70	5.51	.32	<0.001	2.90–10.48	1.70	1.68	1.30 to 4.86
Decompensation + PVT (≤ 3 years)								
ALBI	1.61	5.04	1.50	0.28	0.26 to 9.57	1.61	2.37	–3.09 to 5.98
APRI	–0.36	0.69	0.40	0.36	0.31 to 1.52	–0.36	0.67	–1.95 to 0.74
CHESS-ALARM	0.51	1.66	0.34	0.14	0.84 to 3.28	0.51	0.47	–0.50 to 1.28
CHILD-PUGH	0.83	2.31	0.38	0.02	1.08 to 4.91	0.83	0.87	–1.05 to 2.48
EPOD	–0.48	0.61	0.54	0.37	0.21 to 1.78	–0.48	0.96	–2.16 to 1.6
FIB-4	–0.03	0.96	0.15	0.81	0.71 to 1.30	–0.03	0.27	–0.62 to 0.47
MELD	0.01	1.01	0.08	0.86	0.86 to 1.19	0.01	0.15	–0.28 to 0.35
VITRO	0.01	1.00	0.00	0.19	0.99 to 1.02	0.01	0.03	–0.08 to .066
VAIL	1.69	5.43	0.30	<0.001	2.97 to 9.92	1.69	0.90	1.37 to 4.84
Decompensation alone (≤ 10 years)								
	Variables in the Equation					Bootstrap ^		
**Scores**	β	Exp (β)	se (β)	p	95% C.I. for Exp (β)	β	se (β)	95% C.I. for Exp (β)
ALBI	2.17	8.75	1.54	0.15	0.42 to 17.89	2.17	2.44	–2.68 to 6.17
APRI	0.50	1.66	0.27	0.06	0.96 to 2.87	0.50	0.77	–0.74 to 1.60
CHESS-ALARM	0.34	1.40	0.36	0.34	0.69 to 2.86	0.34	0.44	–0.73 to 1.04
CHILD-PUGH	0.96	2.63	0.33	0.00	1.36 to 5.08	0.96	0.93	–0.33 to 2.16
EPOD	–0.53	0.59	0.51	0.32	0.21 to 1.65	–0.53	0.83	–1.71 to 1.49
FIB-4	–0.36	0.69	0.13	0.00	0.53 to 0.91	–0.36	0.38	–1.13 to -0.03
MELD	0.10	1.11	0.05	0.07	0.99 to 1.24	0.10	0.13	–0.18 to 0.37
VITRO	0.01	1.01	0.00	0.06	0.99 to 1.02	0.01	0.04	–0.10 to 0.03
VAIL	1.30	3.69	0.27	<0.001	2.14 to 6.35	1.30	1.10	0.96 to 3.22
Decompensation + PVT (≤ 10 years)								
ALBI	1.73	5.64	1.49	0.24	0.30 to 10.46	1.73	2.12	–3.08 to 5.35

APRI	0.33	1.39	0.27	0.22	0.81 to 2.38	0.33	0.52	–0.98 to 1.18
CHESS-ALARM	0.21	1.24	0.36	0.55	0.61 to 2.51	0.21	0.48	–1.05 to 0.89
CHILD-PUGH	0.95	2.59	0.32	0.00	1.38 to 4.87	0.95	0.53	–0.15 to 2.03
EPOD	–0.33	0.71	0.51	0.51	0.25 to 1.97	–0.33	0.81	–1.61 to 1.75
FIB-4	–0.27	0.75	0.11	0.01	0.59 to 0.95	–0.27	0.20	–0.78 to 0.06
MELD	0.08	1.08	0.06	0.17	0.96 to 1.22	0.08	0.11	–0.24 to 0.21
VITRO	0.01	1.01	0.00	0.06	0.99 to 1.02	0.01	0.03	–0.07 to 0.03
VAIL	1.27	3.57	0.26	<0.001	2.12 to 5.99	1.27	0.47	0.98 to 2.82

^ Bootstrap were based on 1000 samples.

Abbreviation: β, Coefficient; se (β), Standard Error of β; Exp (β), Exponential of β; 95% C.I., Confidential Interval at 95%.

Table S3 - Baseline predictors of hepatic decompensation occurring in association with portal vein thrombosis.

Cox proportional hazards regression models were used to identify baseline predictors of hepatic decompensation occurring in association with portal vein thrombosis (PVT) during follow-up. Analyses were performed at short-term (≤3 years) and long-term (≤10 years) horizons.

Hazard ratios (HRs) with 95% confidence intervals (CIs) are reported. Variables with $p < 0.10$ in univariable analyses were entered into multivariable models. To minimize collinearity, individual components of composite scores were modeled separately. Multivariable models included clinical variables related to portal hypertension and liver function as well as established prognostic scores. p values were obtained using Wald tests. Statistical significance was defined as $p < 0.05$.

	**≤3-year**				**≤10-year**			
Variables	Univariate		Multivariate		Univariate		Multivariate	
	HR (95% CI)	p	HR (95% CI)	p	HR (95% CI)	p	HR (95% CI)	p
**Model 1**								
Age	1.024	0.021			1.012	0.186		
Sex *	.703	0.158			.665	0.061	.147	0.699
Etiology **	2.227	0.002			1.041	0.268		
BMI	1.020	0.467			1.018	0.395		
Diabetes	.921	0.779			1.488	0.071	1.667	0.041
HVPG	1.116	<0.001			1.099	<0.001		
Vm	.923	<0.001			.924	<0.001		
LSM	1.029	<0.001			1.029	<0.001		
Large Varices	3.771	<0.001	2.270	<0.001	3.761	<0.001	2.858	<0.001
AST	.999	0.835			1.000	0.076		
ALT	.996	0.803			1.000	0.923		
Bilirubin	1.059	0.031	.986	0.708	1.069	0.004	1.004	0.900
Platelets	.985	<0.001			.989	<0.001		
Albumin	.547	<0.001	.498	<0.001	.471	<0.001	.458	<0.001
Creatinine	.933	0.761			.996	0.977		
INR	3.112	0.002	1.664	0.215	3.765	<0.001	3.420	<0.001
CRP	1.384	0.023			1.192	<0.001		

Model 2								
ALBI score	3.771	<0.001	5.049	0.281	2.435	<0.001	5.643	0.245
APRI score	1.106	0.006	.696	0.386	1.163	<0.001	1.393	0.228
CHESS-ALARM	2.046	<0.001	1.666	0.141	2.046	<0.001	1.240	0.550
CHILD-PUGH class	1.892	<0.001	2.312	0.029	4.362	<0.001	2.597	0.003
EPOD score	1.103	<0.001	.617	0.372	1.068	<.001	.714	0.516
FIB-4 score	1.146	<0.001	.965	0.815	1.163	<.001	.757	0.019
MELD	1.123	<0.001	.1.015	0.861	1.123	<.001	1.086	0.177
VAIL score	4.949	<0.001	5.434	<0.001	2.568	<.001	3,571	<0.001
VITRO score	1.002	0.003	1.008	0.194	1.002	.005	1.012	0.999

* Female as reference

** Non viral etiology as reference

Footnote:

Variables reflecting portal hypertension (hepatic venous pressure gradient [HVPG], liver stiffness measurement, portal vein velocity, and presence of large esophageal varices) were highly collinear and were therefore not included simultaneously in multivariable model. Among these variables, large esophageal varices showed the strongest univariable association with hepatic decompensation and was selected for inclusion in the primary multivariable model together with bilirubin, albumin, and international normalized ratio (INR). Hazard ratios (HRs) are reported with 95% confidence intervals (CIs). Supplementary Figure 2

Table S4. **Exploratory association analysis of treatment exposure during follow-up.**

Associations between follow-up treatments, baseline large esophageal varices, and incident hepatic decompensation were assessed using the χ^2 test or Fisher's exact test, as appropriate. This analysis was exploratory and was intended to assess patterns of treatment allocation rather than to estimate the causal effect of treatment on disease trajectory. Treatments were administered according to clinical indications and evolving standards of care. Therefore, significant associations should be interpreted as reflecting indication-driven treatment use and underlying disease severity, rather than independent treatment effects.

Chi-squared test (c2) or Fisher's exact test	Decompensation Follow up (yes/no)	DAAs	Enoxaparin (yes/no)	Aspirin (yes/no)	Beta-blockers (yes/no)	Statin (yes/no)
Large varices baseline (yes/no)	**P=0.001**	P=0.539	P=0.146	P=0.795	**P<0.001**	P=0.388
Statin (yes/no)	P=0.509	P=0.348	P=0.634	**P<0.001**	P=0.056	-
Beta-blockers (yes/no)	**P<0.001**	P=0.705	**P<0.001**	P=0.092	-	-
Aspirin (yes/no)	P=0.629	**P=0.025**	P=0.451	-	-	-
Enoxaparin (yes/no)	**P<0.001**	P=0.076	**-**	-	-	-
DAAs	P=0.096	-	**-**	-	-	--
